# Exceptional Heterogeneity in Viral Evolutionary Dynamics Characterises Chronic Hepatitis C Virus Infection

**DOI:** 10.1371/journal.ppat.1005894

**Published:** 2016-09-15

**Authors:** Jayna Raghwani, Rebecca Rose, Isabelle Sheridan, Philippe Lemey, Marc A. Suchard, Teresa Santantonio, Patrizia Farci, Paul Klenerman, Oliver G. Pybus

**Affiliations:** 1 Department of Zoology, University of Oxford, Oxford, United Kingdom; 2 BioInfoExperts, Thibodaux, Los Angeles, California, United States of America; 3 Peter Medawar Building for Pathogen Research, University of Oxford, Oxford, United Kingdom; 4 Department of Microbiology and Immunology, Rega Institute, KU Leuven–University of Leuven, Leuven, Belgium; 5 Departments of Biomathematics, Biostatistics, Human Genetics, University of California, Los Angeles, California, United States of America; 6 Clinic of Infectious Diseases, University of Foggia, Foggia, Italy; 7 Hepatic Pathogenesis Section, Laboratory of Infectious Diseases, National Institute of Allergy and Infectious Diseases, National Institutes of Health, Bethesda, Maryland, United States of America; Universitat de Valencia, SPAIN

## Abstract

The treatment of HCV infection has seen significant progress, particularly since the approval of new direct-acting antiviral drugs. However these clinical achievements have been made despite an incomplete understanding of HCV replication and within-host evolution, especially compared with HIV-1. Here, we undertake a comprehensive analysis of HCV within-host evolution during chronic infection by investigating over 4000 viral sequences sampled longitudinally from 15 HCV-infected patients. We compare our HCV results to those from a well-studied HIV-1 cohort, revealing key differences in the evolutionary behaviour of these two chronic-infecting pathogens. Notably, we find an exceptional level of heterogeneity in the molecular evolution of HCV, both within and among infected individuals. Furthermore, these patterns are associated with the long-term maintenance of viral lineages within patients, which fluctuate in relative frequency in peripheral blood. Together, our findings demonstrate that HCV replication behavior is complex and likely comprises multiple viral subpopulations with distinct evolutionary dynamics. The presence of a structured viral population can explain apparent paradoxes in chronic HCV infection, such as rapid fluctuations in viral diversity and the reappearance of viral strains years after their initial detection.

## Author Summary

Our knowledge of HCV within-host evolution is substantially limited, which is surprising given that highly successful therapies against the virus have been developed. Key aspects of HCV infection, such as rapid fluctuations in viral diversity and the reappearance of viral strains years after their initial detection, remain unexplained. To better understand this problem, we analyse viral sequences from HCV-infected patients sampled over several years. Our findings suggest that the replication dynamics during chronic HCV infection are distinct from those of HIV-1, and dominated by the co-circulation of multiple viral strains. Although a major difference between the two chronic-infecting viruses is the level of recombination, our results indicate that HCV within-host evolution is most likely to be shaped by a structured viral population. Crucially, our study shows that HCV sampled from blood does not fully represent the within-host viral population at that time. This may have important implications for HCV treatment, especially in patients that have seemingly cleared the virus, as well as for molecular epidemiology studies investigating HCV transmission.

## Introduction

An estimated 3% of the global human population has been infected with the hepatitis C virus (HCV), many of whom are unaware of their infection status. Unlike other members of the virus family *Flaviviridae*, HCV causes acute and chronic infection in humans. Symptoms of acute infection are typically mild and, despite the early response mounted by the immune system, viral clearance occurs in only 15–20% of untreated cases. In the remaining individuals who become chronically-infected, the virus can, over many years, cause liver cirrhosis, hepatocellular carcinoma, and other related diseases. Genetically, HCV is a very diverse virus and up to 50% of nucleotide sites may vary among HCV strains belonging to different genotypes. The high genetic diversity of HCV is the product of both a high rate of molecular evolution and a proposed long-term association of the virus with human populations [1].

Prior to the discovery in 2003 of an atypical HCV genotype 2 strain that can readily replicate in hepatoma cell lines [2], the development of HCV-specific antiviral drugs was comparatively slow and, until recently, standard drug treatment for HCV infection was non-specific and involved long courses of interferon and ribavirin. However, newly approved direct-acting antiviral (DAA) drugs that target the HCV life cycle are highly effective, leading to viral clearance in >90% of patients within 12 to 24 weeks of treatment [3–7]. Interestingly, these clinical successes have been achieved despite relatively little being known about the *in vivo* dynamics of HCV replication, host cell infection, and evolution.

Most of our understanding of HCV replication behaviour within infected individuals has come from mathematical models of virus kinetics [8], which are typically fitted to measurements of viral load from longitudinal samples of peripheral blood. Simple models that employ a mass action mechanism of infection can explain the two-phase decline in HCV viral load following interferon-based drug therapy and have demonstrated (i) a high turnover of virions in peripheral blood [9], (ii) a high variance among patients in the mean lifespan of infected cells, ranging from 2–70 days [9], and (iii) that approximately 3% of virions in serum result from extra-hepatic replication [10]. More complex viral load dynamics, including triphasic decline and the failure of drugs to fully eradicate the virus, have been explained by adding proliferation of infected and non-infected hepatocytes to the model [11]. However, it is unclear whether division of infected hepatocytes requires active virus replication, or whether HCV is passively transferred between parent and daughter cells.

An alternate hypothesis, that HCV persists in exceptionally long-lived cells during chronic infection, has been discounted [11, 12]; yet it is known that uninfected hepatocytes have a significantly slower turnover than the main target cells of HIV (CD4+ T lymphocytes) and are thought to survive for years [13]. Further, despite the important insights revealed by mass-action models of HCV virus kinetics, they cannot fully reconcile all aspects of chronic HCV infection *in vivo*. This includes observations of cell-to-cell virus transmission [14–16], foci of infection within the liver [17–21], and viral re-emergence after drug therapy has temporarily reduced viremia in peripheral blood to undetectable levels [22–25].

Standard models of virus infection kinetics were initially developed in the context of HIV-1 infection [26, 27], in which virions sampled from peripheral blood appear to be representative of the contemporaneous population of actively-infected host cells. Whether this assumption is also true for HCV is difficult to ascertain because sampling of liver tissue, the primary site of replication, is invasive and rarely repeated longitudinally during infection. Molecular and mathematical analysis of individual liver biopsy samples indicates that HCV infection spreads locally within the liver and is likely to be seeded randomly by viruses from peripheral blood [28]. Further, analysis of virus gene sequences obtained from transplantation patients and from explant livers suggests that hepatic and extra-hepatic viruses can be genetically distinct and may form different sub-populations [29–32]. Recent experimental and clinical studies have suggested a more complex model of HCV replication, involving cell-to-cell transmission dampened by localized immune responses, as well as detectable virus replication in quiescent hepatocytes (i.e. cells that are differentiated but in a resting state) and non-hepatic reservoirs [10, 33]. Importantly, these observations suggest that viral replication dynamics during HCV infection may be decoupled, at least in part, from host cell turnover.

Viral gene sequences, sampled longitudinally through time from chronically infected patients, constitute a valuable and independent source of information about replication dynamics. The high mutation of HCV means that viral genomes accrue ~0.3 to 1.2 nucleotide substitutions per cell infection [34, 35]. As a consequence, the genetic divergence between viruses sampled throughout infection will be influenced by both the mode and tempo of cell-to-cell infection. Investigation of longitudinally sampled virus sequences has proven useful for HIV-1 infection, leading to insights regarding the size of viral bottlenecks at transmission [36–38], correlations between viral evolution and clinical outcomes [39, 40], and the relationship between within- and among-host virus evolution [41]. Studies of serially-sampled HCV sequences have also indicated a link between viral evolution and disease progression. First, the level of HVR1 diversity during acute infection has been associated with whether a patient successfully clears the virus [42]. Second, greater genetic diversity and synonymous divergence is observed in viral populations sampled from rapid progressors, which suggests that faster disease progression is associated with shorter viral generation times [43], as has also been reported for HIV-1 [40]. Nonetheless, these observations are based on studies with limited number of patients and viral sequences, and which used only simple summary statistics (e.g. pairwise diversity) during analysis.

To better understand the replication dynamics of HCV during infection, we undertake a comprehensive analysis of HCV evolutionary dynamics during chronic infection. We use statistically powerful Bayesian phylogenetic approaches to test hypotheses concerning the diversity and divergence through time of within-host HCV populations. In total, we analyse more than 4000 viral gene sequences obtained from 15 patients, sampled over 100 different time points. We compare our HCV results to those obtained from nine comparable HIV-1 infected subjects, and discover differences between the evolutionary dynamics of the two viruses during chronic infection. Most notably we observe significant heterogeneity in the molecular evolution of HCV, both among patients and over time, which contrasts with more consistent trends in HIV-1 infected patients. Our results support a complex model of HCV replication dynamics during chronic infection that reconciles apparent paradoxes observed in the natural history of this infection

## Results

### Per sample summary statistics

**Fig 1:**
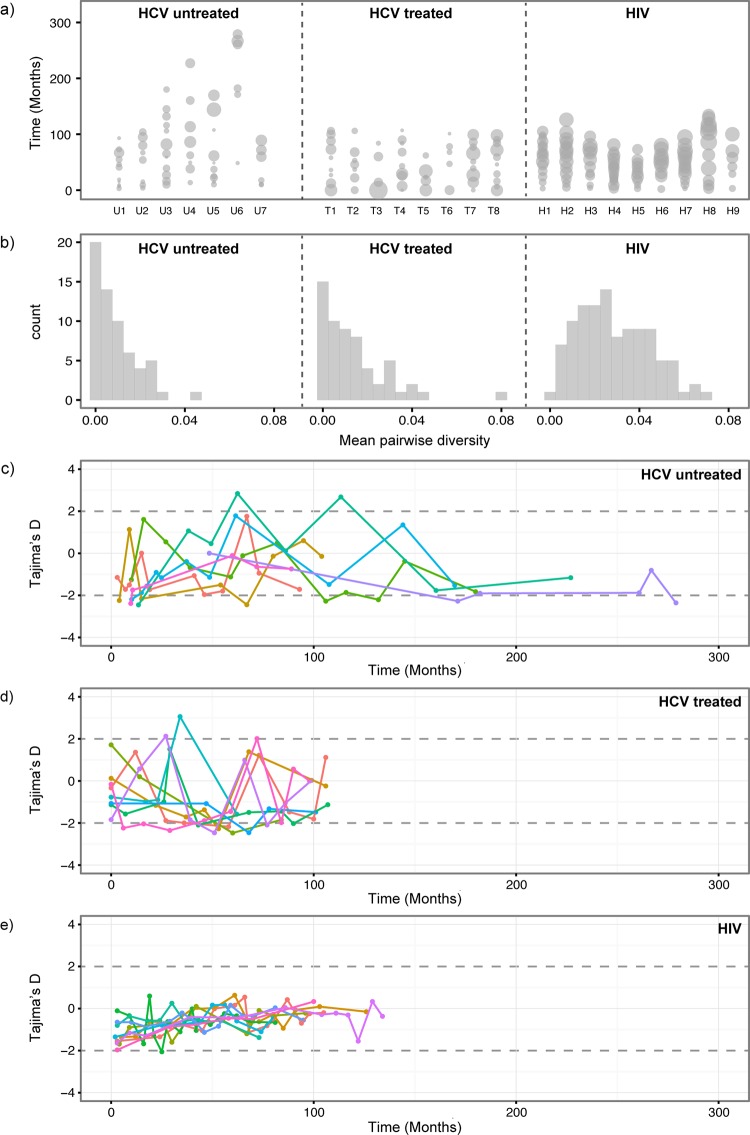
Per-sample summary statistics. (a) Mean pairwise nucleotide diversity (MPD) for each time-point and each subject. The relative width of each circle represents MPD. Each column represents the values for one subject (U1-7, T1-8 and H1-9). The y-axis show the time of sampling. For subjects U1-7 and H1-9, time zero is the known (or closely estimated) date of infection. For subjects T1-8, time zero equals the date of the first sample. (b) The distribution of MPD values is shown for each group of patients. Panels (c), (d) and (e) show Tajima’s D estimates for each time-point and each subject. The different colours indicate different patients. The estimates above 2 and below -2 (indicated by dashed horizontal lines) correspond to significant deviation from neutrality. (c) Untreated HCV subjects U1-7. (d) Treated HCV subjects T1-8. (e) HIV subjects H1-9.

The amount of diversity among viruses sampled at each time point is shown in Fig 1A, where the size of each circle is proportional to the mean pairwise sequence diversity (MPD) for that time-point. If we average the MPD scores across all subjects and time points then we obtain 0.009 changes/site for the HCV untreated group and 0.013 changes/site for the HCV treated group. The overall genetic diversity is higher for HIV-1 patients (average MPD across all time points = 0.029). We also found interesting differences between HIV-1 and HCV patients in the distribution of viral diversity among time points. Specifically, we find that the distribution of MPD scores for the HIV-1 group is much more symmetric (skewness = 0.37) than for the two HCV cohorts (skewness = 1.38 and 2.08, for HCV treated and untreated subjects, respectively; Fig 1B). The strong positive skew observed for both HCV groups indicates that, during infection, HCV exhibits more extreme occasional shifts to high viral diversity, despite the fact that, on average, viral population diversity is low compared to HIV-1 infections. Treatment periods (interferon and ribavirin) in the HCV treated group do not appear to correlate with lower genetic diversity, although this cannot be formally tested because the relative timing of sampling times and treatment periods varied among subjects.

To characterize change in the genetic structure of the within-host viral population we calculated Tajima’s D statistic for each time point in each patient (Fig 1C–1E). This statistic varies significantly over the course of infection in HCV patients, with rapid fluctuations even between immediately adjacent time points (Fig 1C and 1D). This demonstrates substantial changes in the frequency distribution of polymorphic sites. In other words, the viral population shifts back and forth between carrying many common polymorphisms (D>0) and carrying many unique low-frequency variants (D<0). In contrast, the genetic structures of within-host HIV-1 populations are more stable through time and predominated by rare or low-frequency polymorphisms (D<0; Fig 1E). For HIV, Tajima’s D statistic gradually rises through time but rarely exceeds zero (Fig 1E). Consequently, when comparing the distributions of Tajima’s D values among the three cohorts, for both untreated and treated HCV patients we observed significantly greater variance and positive skew in Tajima’d D values compared to HIV-1 patients (S1 Fig). In addition, for all groups of subjects, Tajima’s D values for each time point are positively correlated with viral genetic diversity (S2 Fig; p<0.001 for all three groups; correlation test), such that when diversity is low, shared mutations are more likely to be rare. Theory predicts both MPD and Tajima’s D values will be low when a sampled population has recently experienced an expansion, either due to rapid population growth or a recent selective sweep. High values of both statistics are predicted when population structure or fluctuating selection maintains genetic diversity in a population.

### Rates of viral molecular evolution

**Fig 2:**
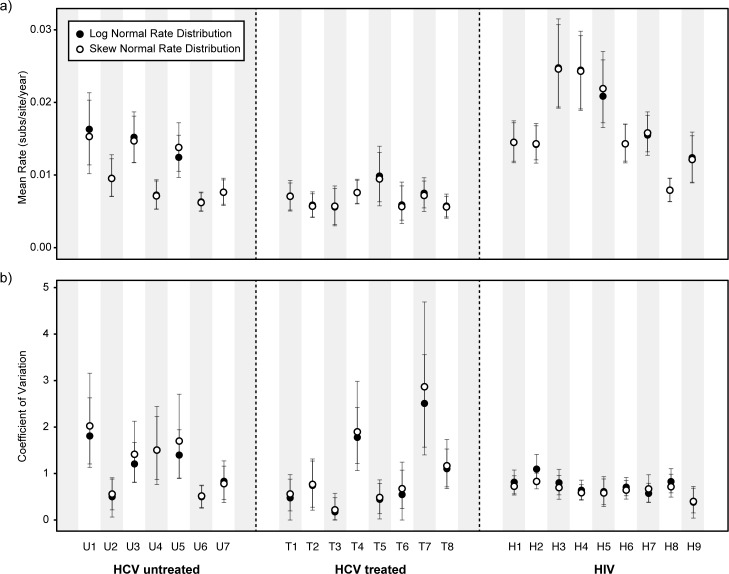
Estimation of evolutionary rates. (a) Estimated mean viral evolutionary rate for each subject in the three groups (HCV untreated, HCV treated, and HIV). (b) Estimated coefficient of variation (COV) of the relaxed molecular clock, for each subject. Filled circles indicate estimates obtained under a molecular clock with a log-normal distribution of among-branch rates. Open circles indicate estimates obtained under a molecular clock with a skew-normal rate distribution of among-branch rates. The 95% highest posterior density (HPD) intervals for each estimate are indicated by vertical error bars.

The mean rates of molecular evolution for each subject, as estimated using the lognormal relaxed molecular clock model, are shown in Fig 2A. The mean rate is notably lower in drug-treated HCV subjects than in the HCV untreated group (Fig 2A; Mann-Whitney U test, *p* < 0.05). The evolutionary rate is in general higher for HIV-1 than for HCV (we place no emphasis on this comparison because the HIV-1 and HCV genome regions are not homologous). Fig 2B shows, for each patient, the degree to which the viral evolutionary rate varies during infection, which is quantified using the coefficient of variation (COV) of the relaxed molecular clock. Two patterns are evident. First, the COV statistic is more variable among HCV subjects than among HIV-1 subjects. Second, extremely high levels of viral rate variation are observed in some HCV subjects, but not in HIV-1 subjects (estimated COV>1 for seven HCV patients, but only one HIV-1 patient). The values in some HCV subjects are unusually high (COV>1.75) and represent exceptional rate variation among lineages (Fig 2B). To test whether these estimates were robust to model misspecification, we implemented a new relaxed molecular clock that assumes that branch rate scalars follow a more flexible skew-normal distribution. Unlike the standard lognormal molecular clock, the skew-normal molecular clock allows the distribution of evolutionary rates among branches to be either positively or negatively skewed, or non-skewed. Both the skew-normal and lognormal molecular clocks give similar parameter estimates (Fig 2; filled and open circles indicates estimates under log-normal and skew-normal rate distribution, respectively). Furthermore, the shape parameter of the skew normal model differed significantly from zero in only one patient (S3 Fig), indicating that the distribution of among-branch rate variation was approximately symmetric.

**Fig 3:**
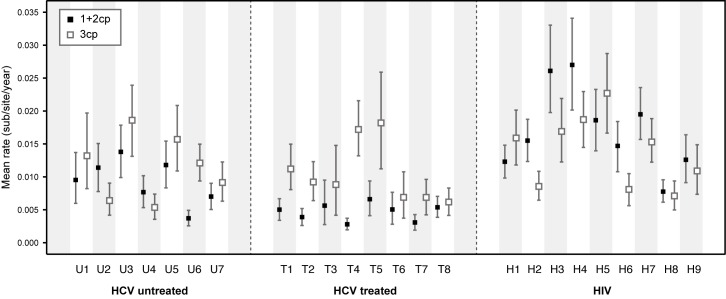
Estimation of evolutionary rates for codon partitions. Evolutionary rates for the two codon partitions (1+2 cp and 3cp) were estimated separately for each subject. Black squares indicate the mean evolutionary rate for 1+2cp sites, and white squares indicate the mean evolutionary rate for 3cp sites. The 95% HPD intervals for each estimate are indicated by vertical error bars.

To explore why rates of molecular evolution are lower in the HCV treated group than in the untreated group (Fig 2A) we used a partition model to estimate rates of evolution for first and second codon positions (1+2cp) and third codon positions (3cp; Fig 3). These rates contain information about the action of positive and negative selection because the majority of mutations at 1+2cp and 3cp sites are, respectively, non-synonymous and synonymous. This approach is a good proxy for dN/dS values estimated with codon substitutional models, which for large temporally sampled datasets can be difficult to obtain due to slow MCMC convergence. However, we note that, unlike dN/dS ratios, the ratio of codon position rates cannot be used to formally test for positive selection (Table 1).

**Table 1:** Ratio of 1+2cp and 3cp rates for each patient from the three different patient cohorts.

Patient	Ratio	Group
U1	0.72	HCV untreated
U2	1.77*	HCV untreated
U3	0.74	HCV untreated
U4	1.43*	HCV untreated
U5	0.75	HCV untreated
U6	0.31	HCV untreated
U7	0.77	HCV untreated
T1	0.45	HCV treated
T2	0.42	HCV treated
T3	0.64	HCV treated
T4	0.16	HCV treated
T5	0.36	HCV treated
T6	0.73	HCV treated
T7	0.45	HCV treated
T8	0.87	HCV treated
H1	0.77	HIV
H2	1.81*	HIV
H3	1.55*	HIV
H4	1.44*	HIV
H5	0.82	HIV
H6	1.81*	HIV
H7	1.28*	HIV
H8	1.09*	HIV
H9	1.16*	HIV

Ratios above 1 are indicated by an asterisk (*), which is suggests higher non-synonymous rate relative to the synonymous rate.

Amongst HCV subjects, 3cp rates (open squares) are largely similar between the treated group and untreated group, whereas the 1+2cp rates (filled squares) are lower in HCV subjects that have received treatment (Mann-Whitney U test, p <0.01; Fig 3). Thus the reduced overall rate of virus evolution (Fig 2A) in the HCV treated group appears to be caused by reduced evolution at 1+2cp sites (Fig 3), suggesting that drug-therapy has reduced the ability of the viral population to undergo adaptive fixation (Table 1), but has not significantly reduced the fixation of 3cp changes that are likely to be selectively neutral (see also S4 Fig). In contrast to HCV, seven of the HIV-1 subjects had a higher estimated evolutionary rate at 1+2cp sites than at 3cp sites (Table 1 and Fig 3), indicating greater positive selection and/or less negative selection on the HIV-1 sequences. Many previous studies have demonstrated adaptation of the HIV-1 *env* gene during infection due to positive selection (e.g. [39, 44, 45]). For HIV-1 subjects, both the 1+2cp and 3cp rates are correlated with total evolutionary rate, whereas for HCV subjects, only the 1+2cp rate exhibits such a correlation (S4 Fig).

### Phylogenetic structure and population genetic diversity

**Fig 4:**
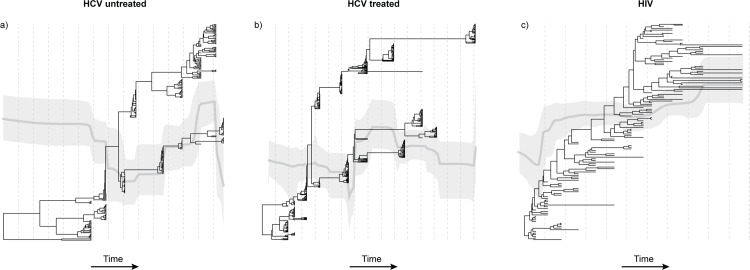
Phylogenetic structure. A maximum clade credibility phylogeny is shown for one representative subject from each patient group (HCV untreated, HCV treated, and HIV). Branches are scaled by time. Superimposed on each phylogeny, on the same timescale, is the estimated Bayesian skyline plot for that subject. The light grey line indicates the mean skyline plot estimate of effective population size through time. The darker grey areas indicate the 95% highest posterior density credible interval for that estimate. The distance between dotted vertical lines indicates one year. (a) Patient U3 from the HCV untreated group. (b) Patient T1 from the treated HCV group. (c) Patient H2 from the HIV cohort.

There are several notable differences between the estimated time-scaled phylogenies from HCV subjects compared to those from HIV-1 subjects. One representative phylogeny from each patient group is shown in Fig 4, and all phylogenies are presented in S5–S7 Figs. The vertical dashed lines indicate yearly intervals in each patient phylogeny. Firstly, during HCV infection distinct lineages can persist for extended periods of times; in Fig 4A and 4B this can be between 7 and 9 years, respectively. To quantify this we calculate the ratio of external to internal branch lengths for the two HCV phylogenies in Fig 4. The mean ratios are significantly less than one: 0.49 (95% HPD = 0.38,0.59) for the untreated HCV patient and 0.56 (0.48, 0.66) for the treated HCV patient. In contrast the mean ratio for the HIV-1 phylogeny is 1.94 (1.65, 2.25), indicating that viral lineage turnover is faster (Fig 4C). Further, the persistent lineages observed in HCV infection may go undetected for many years; hence the number of divergent lineages that are actually detected at any given sampling time may vary.

Secondly, HCV sequences sampled from the same time-point on the same lineage tend to share a very recent common ancestor, giving rise to a distinctive phylogenetic pattern of long internal branches punctuated by ‘bursts’ of closely related or identical sequences. When only a single lineage is sampled at a given time point, this leads to a low observed MPD and a strongly negative value of Tajima’s D. This indicates that all the HCV sequences belonging to that lineage represent a viral subpopulation that has recently expanded or been subjected to a recent population bottleneck. However, when multiple HCV lineages are observed at a given time-point, then the sample MPD for that time-point is, by definition, higher and the corresponding Tajima’s D is typically closer to zero or positive. This association between phylogenetic structure and genetic diversity explains both the results for HCV in Fig 1 and the correlation between MPD and Tajima’s D (S2 Fig). In comparison, sequences sampled from a given time-point during HIV-1 infection share a comparatively recent common ancestor on the persistent ‘backbone’ of the phylogeny (Fig 4C). Furthermore, the high ratios of external to internal branch lengths in the HIV phylogenies are expected by theory if the viral population is unstructured and undergoing recurrent selective sweeps. This result explains the consistently negative Tajima’s D values and the steady changes in MPD observed for HIV-1 in Fig 1.

Changes in relative population genetic diversity during infection are illustrated by Bayesian skyline plots, which are superimposed over the phylogenies in Fig 4 (the timescale of the skyline plots and phylogenies are shared). Note that the skyline plot represents the total diversity of the entire within-host viral population through time, including lineages that are inferred to be present but unsampled, whereas the MPD values in Fig 1 represent only the diversity that is actually sampled at each time point. There are no clear trends among patient groups in the dynamics of viral population diversity, although significant declines are perhaps more common in the HCV treated group than in either of the untreated groups (S5–S7 Figs).

## Discussion

Patterns of viral genetic divergence and diversity during chronic infection depend on the structure and dynamics of the replicating viral population, and therefore they provide a source of information about infection kinetics that is independent from and complementary to mathematical models of longitudinal viral load measurements [8–11]. Further, molecular clock approaches like those used here may better resolve complex evolutionary dynamics than analyses of sequence summary statistics, which uses data less efficiently [46]. The results of our evolutionary analyses show that intra-patient HCV evolution is exceptionally heterogeneous, both within and among different subjects, compared with intra-patient HIV-1 evolution, and that this variation is present in both treated and untreated HCV-infected subjects. Specifically, for HCV we find (i) extreme heterogeneity in the rate of molecular evolution in some patients; (ii) a lower rate of non-synonymous change in patients that received interferon-treatment; (iii) significant fluctuations in viral genetic diversity through time; and (iv) unusual phylogenetic topologies containing multiple distinct lineages that coexist for long periods of time, combined with ‘bursts’ of closely-related sampled variants. These observations are not consistent with a well-mixed viral population with homogenous infection dynamics, but instead suggest that HCV infections are comprised of multiple sub-populations with distinct evolutionary and replication behaviours.

While rates of HCV molecular evolution *in vivo* are comparable to those estimated for other RNA viruses (e.g. HIV-1 and influenza) [47], we observe very high among-lineage rate heterogeneity only for some HCV infections. This suggests, at the very least, that circulating HCV lineages do not all accumulate substitutions in the same manner. Rates of viral sequence divergence are determined by mutation rates, population sizes, generation times, and mutational selection coefficients. Crucially, the latter three factors can only vary within an individual if the within-patient viral population is split into distinct subpopulations with separate dynamics.

There is a growing body of independent evidence that indicates the presence of an HCV population structure in the liver. The existence of genetically distinct viral sub-populations (compartmentalization) has been demonstrated for viruses isolated from (i) plasma versus liver [29, 30, 48–53], (ii) different locations within the same liver [32], and (iii) between non-tumourous liver tissue versus tumour-associated liver tissue [32, 54–57]. Experimental studies demonstrate that, within the liver, HCV tends to be localized to specific foci of infection [18]. If cell-to-cell transmission is more efficient than transmission via free virions, then models of HCV infection should incorporate local viral replication, adaptation and spread within the organ [58]. Viral population structure may also exist outside the liver, as HCV genetic compartmentalisation has been reported (i) among cirrhotic liver samples [50], (ii) between plasma and PBMCs [29, 30, 49, 53, 59–61], (iii) between PBMCs and the liver [29–31, 49, 59], (iv) between liver and perihepatic lymph nodes [53], and (iv) in the brain [62].

The heterogeneity in HCV evolutionary rate we report here is consistent with these experimental results, and we posit that it arises from distinct sub-populations of HCV (hepatic or extra-hepatic) whose replication is modulated by local fluctuations in host cell availability and turnover, and/or by anti-viral immune responses. The modulation of replication within separate viral sub-populations can also readily explain the unusual HCV phylogenetic topologies. Specifically, HCV lineages that are present in the body, but which are not directly observed in peripheral blood for prolonged periods of time, might represent sub-populations that are not shedding virions into circulation, either because they are replicating slowly, or because they are transmitting via cell-to-cell contact. Cell-to-cell transmission may allow HCV to replicate in the presence of neutralizing antibodies [14, 58] and is again consistent with the detection of hepatic foci of infection [18, 20]. One recent study has found that *in vitro* DAA drug-resistant viruses predominantly spread by this route of transmission [16]. Further, the ‘bursts’ of closely related sequences that we observe are consistent with the recent and rapid growth of previously restricted viral subpopulations. The causes of these bursts are unknown; possible explanations include viral adaptation or the local deterioration of immune control.

Viral population structure and host immune responses could also account for the puzzling fact that most cells in the liver are uninfected [18, 35, 63]. This is surprising given that viral loads in serum are high (10^5^–10^7^ virions/mL), and that transplant livers are rapidly re-infected following transplantation. If the viral population is strongly structured then chronic infection requires only the establishment of a few long-lasting sub-populations that are not removed by host immune responses. It is not known whether the distinct lineages observed during HCV infection are antigenically distinct. If they are, this antigenic variation may contribute to the creation and maintenance of a persistent infection.

The highly structured nature of HCV intra-host genetic diversity also has consequences for the evolutionary analysis of chronic infection. Specifically, it means that samples of HCV diversity from peripheral blood do not adequately characterise the genetic diversity of the infection as a whole [46]. We find that statistics of sample diversity (MPD and Tajima’s D) vary substantially through time within HCV subjects, but are more consistent (Tajima’s D) or less skewed (MPD) for HIV-1 subjects. Molecular clock phylogenetic analyses show that this is due to significant among lineage rate variation. Whilst PCR primers might fail to amplify some within-host HCV lineages, it is difficult to conceive how differential amplification might cause strong fluctuations in viral diversity through time *within* a single patient.

Given that the number of sequences per time point in our data sets is comparatively small (range n = 18 to n = 88) it could be argued that the intermittent detection of HCV lineages in peripheral blood is solely a consequence of sampling uncertainty. To explore this, let us suppose there are two lineages, in which case the probability of detection can be determined by the binomial distribution. If n = 40 and sampling is random, then a lineage whose population frequency is 5% will be detected at 87% of timepoints, but a lineage whose frequency is 0.5% will be seen at only 4% of timepoints. Lineages at frequencies between ~0.5% and ~5% are therefore likely to be intermittently detected in our study. However the key observation that HCV lineages are often dominant at one timepoint, but rare or absent at a later timepoint, is not an artefact of sampling uncertainty because the sample sizes used in our study will almost certainly detect all lineages whose frequencies exceed 15%. We also note that the evolutionary patterns in HCV subjects reported here have come from different cohorts generated using different sequencing approaches, and similarly structured within-host HCV phylogenies have been noted elsewhere [64–66]. Although we cannot pinpoint the anatomical locations of HCV genetic sub-populations, these are likely to be sites within the liver and/or extra-hepatic compartments such as PBMCs or the central nervous systems [30, 62]. Cross-sectional genetic analysis of HCV diversity in explanted livers may help to address this question.

Low recombination in HCV [67] could also potentially explain differences between the within-host molecular evolution of HIV-1 and HCV. Specifically, infrequent recombination can lead to stronger clonal interference, whereby beneficial mutations on different genetic background compete for fixation [68], resulting in longer times to fixation of mutations and increased diversity at each sampling time. While this effect is likely to shape HCV molecular evolution, and may increase the length of some internal phylogenetic branches, it cannot account for the alternating appearance of divergent lineages in peripheral blood after long periods of absence. Further, low recombination would lead to complete selective sweeps and is therefore inconsistent with the long-term persistence of multiple lineages (e.g. > 20 years in one treated HCV subject; see S6C Fig) observed in our HCV cohorts.

The hypothesis that strong HCV population structure and lineage rate variation contributes to viral persistence has consequences for the new DAAs that are highly successful in treating HCV infection. Although these treatments drastically reduce treatment times, a longer-follow up of patients may be prudent if there is a longer-term risk of viral relapse from unsampled reservoir populations within the body. A recent study has found that viral persistence is prevalent in patients who have spontaneously resolved the virus [69]; HCV RNA was detected in ~70% of patients ~6 years after clearing the virus. Furthermore, samples collected from PBMCs between 5 to 20 years after initial detection of HCV supports ongoing viral replication despite patients appearing non-viraemic [69]. Very late HCV breakthroughs have been reported from some clinical trials using DAA therapy [23–25]. Although these instances are infrequent, they do highlight that our understanding of the persistence of HCV at low levels is inadequate and requires further investigation.

Lastly, the high evolutionary heterogeneity of HCV within hosts has important implications for molecular epidemiological analyses of HCV genetic diversity at the among-host level. In such studies each infected individual is typically represented by a single sequence that is interpreted as the ‘consensus’ of the within-host viral population at the time of sampling. For HCV, the intermittent detection in sera of diverse lineages means that the consensus sequence obtained may be highly dependent on when sampling occurs, and may not be representative of the virus that is transmitted. Crucially, this could explain in part why among-host HCV molecular clock phylogenies have proven difficult to calibrate from longitudinal samples of HCV sequences [70].

## Materials and Methods

### Datasets

We analysed a total of 15 HCV infected subjects. Subject and sampling information is provided in Tables S1-3. Subjects from previously published studies were only included if HCV sequences were sampled longitudinally for at least 5 years. HCV sequences were obtained from seven untreated patients previously reported by [43, 71] (referred to as U1-U7 in this study). These subjects acquired HCV infection either perinatally (U1-3) [43] or via transfusion (U4-7) [71]. The date of infection was known and thus all time points represent time since infection. To enable direct comparison with other subjects, HCV sequences sampled during acute infection were removed (U1, U4, U6: time points <3 months; U5: time points <9 months; U7: time points <8 months). The sequences represent partial E1/E2 gene sequences corresponding to positions 1308–1835 relative to the H77 HCV reference genome. Alignments from these patients included a total of 2246 sequences (range 235–418 sequences per subject) and an average of 8.7 time points per subject (range 6–12 time points) that cover an average duration of sampling of 13.6 years (range 7.4–23.3 years).

Sequences from an additional 8 subjects were obtained from sequential serum samples from a cohort of HCV patients from Bari, Italy. The hypothesised route of transmission was nosocomial infection following surgery: no other risk factors were observed and all patients were anti-HCV negative at the time of surgery, however none of them received a blood transfusion. These subjects (denoted T1-8) were treated with interferon and ribavirin; all subjects received at least one period of therapy during the study, although duration and regimen varied among subjects. Sequences from these subjects were generated by amplifying segments of the E1/E2 gene region using multiple different primer pairs that spanned the hyper-variable region 1 (HVR1). Full sequencing details for this cohort can be found in Supporting Information (S1 Text). Sequences were trimmed to match those obtained from patients U1-7 and corresponded to positions 1320–1799 relative to the H77 HCV reference genome. At least 18 clonal sequences were generated per time-point. Alignments from subjects T1-8 included a total of 1980 sequences (range 132–395 per subject), with an average of 7.3 time points per subject (range 4–10) covering an average of 7.9 years of infection (range 5.2–8.8 years). The HVR1 region was targeted for sequencing in both untreated and treated HCV cohort as it is the most diverse region in the HCV genome, and consequently contains the strongest phylogenetic signal compared to other, more conserved genomic regions. A comparable set of previously published sequences from a cohort of untreated HIV-1 infected subjects (HIV1-9) was analyzed concurrently [72]. All subjects were infected with subtype B and sequences represented the C2-V5 region of the gp120 gene (corresponding to positions 7023–7286 in the HXB2 HIV reference genome). The total number of HIV-1 sequences was 1028 (range 52–160 per subject), with an average of 11.7 time points per subject (range 6–15) spanning an average of 8.2 years of infection (range 6.1–11.2).

To verify and subtype the HCV sequences, an alignment was created containing the HCV sequences from all 15 subjects, plus reference sequences from each of the major HCV subtypes and genotypes. A neighbour-joining tree was reconstructed under the HKY nucleotide substitution model using MEGAv5.0 [73]. Two hundred bootstrap replicates were used to assess the robustness of the tree topology. Sequences from each subject clustered with each other, and not with sequences from other subjects, with high bootstrap support. In the untreated cohort, subjects were singly infected with subtypes 1a, 1b, and 4d, while in the treated cohort all patients were infected with subtype 1b.

### Per-sample summary statistics

The genetic diversity of the intra-host viral population at each time point in each subject was estimated by calculating mean pairwise genetic distances among sequences using a Tamura-Nei substitution model with gamma distributed rates, as implemented in MEGA5.0 [73].

We also calculated Tajima’s D statistic for each sampling time in each subject, using DNAsp [74]. Tajima’s D statistic describes the relative frequency of common versus rare polymorphisms in the sample, and consequently describes whether the sample phylogeny is star-like (long external branches) or structured (long internal branches). Tajima’s D is expected to be zero under a null model of constant size population with no natural selection or population structure. Negative D values indicate an excess of rare polymorphisms compared to this null model, which may result from a recent selective sweep or population growth. Positive D values indicate an excess of common polymorphisms, which may be caused by population contraction, or population structure, or by fluctuating selection.

### Estimation of evolutionary rates

Rates of within-host molecular evolution (divergence rates) were investigated using the Bayesian Markov chain Monte Carlo framework implemented in BEAST v.1.8 [75]. An initial set of model selection analyses were undertaken to explore different coalescent and molecular clock models (in each case the codon-structured SDR06 nucleotide substitution model was used). Simple coalescent models (constant size and exponential growth) failed to converge for some HCV datasets, so final analyses were performed using the Bayesian Skyline coalescent model. Preliminary analyses indicated significant among-branch rate heterogeneity so a relaxed uncorrelated molecular clock was used. Analyses were first performed using the standard log-normal distribution model, for which the among-branch rate distribution is negatively skewed. However, we were concerned that this model may not adequately capture the rate variation in within-host HCV evolution. Therefore we also implemented a new molecular clock model in BEAST 1.8 with a skew-normal distribution of among-branch rate variation, which allows the among branch rate distribution to be either positively or negatively skewed, or unskewed (see S2 Text for example XML code). Evolutionary rates were also estimated separately for (i) combined 1^st^ and 2^nd^ codon positions (1+2cp) and (ii) 3^rd^ codon positions (3cp), using a log-normal molecular clock model. MCMC convergence was generally slow and chain length varied between 100–200 million generations. Chains were sampled regularly to yield 10000 samples. Multiple independent runs were undertaken to ensure adequate mixing and stationarity had been achieved, as diagnosed using trace plots and effective sample sizes.

### Exploring the potential effects of sequence undersampling

Our historical data sets were generated using clonal Sanger sequencing and contain far fewer sequences per time point (n = 18–88) than could be generated using modern next-generation sequencing (NGS) platforms (100s or 1000s of sequences per time point). To explore the potential effects of this on our estimates of statistics of viral genetic diversity, we simulated the process of undersampling upon previously published NGS datasets for both chronic HIV and HCV infections. We looked for NGS within-host data sets within which we could identify non-overlapping regions of varying genetic diversity that were 350-400nt length and which were represented at depth of 500 reads or greater. Suitable HIV data was found in Zanini et al [76] and Dialdestoro et al [77], and comparable HCV data was obtained from Lu et al [78].

We randomly subsampled these NGS datasets to simulate the effects of undersampling. Specifically, in each case, we generated 100 randomly subsampled datasets containing 5, 10, 12, 14, 16, 18, 20, 40, 60, 80, and 100 sequences. For each replicate subsample we estimated mean MPD and Tajima’s D in exactly the same way as for the real data (see above). These results are summarized in S8 and S9 Figs.

In all cases, the variability and uncertainty in estimates of MPD and Tajima’s D drops quickly as sample size (n) increases above 10. In our data sets, sample sizes per timepoint range from n = 18 to n = 88 (shown in S8 and S9 Figs as red dashed lines). In this range of sample sizes, estimates of MPD and Tajima’s D are close to those obtained from the full (non-subsampled) dataset. In general, variance in estimates of these statistics stabilises between n = 5 and n = 18 sequences, and this is seen in both low and high diversity genome regions. This indicates that our estimates of MPD and Tajima’s D (Fig 1) are very similar to those that would be obtained from NGS data sets comprising hundreds or thousands of reads, and that the observed variation in these statistics among time points is not due to sampling uncertainty (or small sample sizes); instead the variation is due to real changes in the viral population. While NGS datasets would undoubtedly reveal many more rare variants, such variants have very little effect, by definition, on statistics that summarise the genetic composition of the population as a whole.

### Ethics statement

HCV isolates were obtained from adult patients with diagnosis of acute hepatitis C followed at the Clinic of Infectious Diseases, University of Bari. The study was approved by the local Ethical Committee (EC University of Bari) and a written informed consent was obtained from each patient.

## Supporting Information

S1 Fig Distribution of Tajima’s D values for three cohorts. The distributions of Tajima’s D values are plotted for each cohort (HCV untreated, HCV treated, and HIV-1). The HCV untreated and treated groups have very similar distribution of Tajima’s D values, which show greater positive skew and variance compared to HIV-1 patients.
(PDF)

S2 Fig Correlation between Tajima’s D and mean pairwise nucleotide diversity (MPD). Tajima’s D (x-axis) is plotted against pair-wise nucleotide diversity (y-axis) for all time-points and subjects. (a) HCV untreated group; (b) HCV treated group; (c) HIV-1 group. A fitted linear regression model and associated R^2^ value are shown for each group.
(PDF)

S3 Fig Mean estimate of the shape parameter of the skew-normal relaxed molecular clock model. Mean estimate (filled circle) and 95% confidence intervals (vertical bars) of the shape parameter (y-axis) are shown for all subjects (x-axis).
(PDF)

S4 Fig Correlation among evolutionary rates estimated from different alignment partitions. Mean estimate of the evolutionary rate for first and second codon positions (y-axis, both panels) is plotted against (a) the total mean evolutionary rate for all sites, and (b) the mean evolutionary rate for third codon positions. Each point represents a different subject. Subjects in the untreated HCV group are shown as open circles, those in the treated HCV group as filled circles, and those in the HIV-1 group as red squares. A fitted linear regression model and associated R^2^ value are shown for HCV and HIV-1 groups.
(PDF)

S5 Fig Demographic history of untreated HCV subjects. The maximum clade credibility tree and skyline plots are shown each subject group. Panels (a) to (g) represent subjects U1-7, respectively. See main text Fig 4 for more details. Trees and skyline plots were inferred using the log-normal relaxed molecular clock model. Dotted vertical lines indicate one year and branches are scaled by time. The light grey line indicates the mean population diversity estimates through time, and the darker grey areas indicate the 95% HPD intervals of that estimate.
(PDF)

S6 Fig Demographic history of treated HCV subjects. The maximum clade credibility tree and skyline plots are shown each subject group. Panels (a) to (h) represent subjects T1-8, respectively. See main text Fig 4 for more details. Trees and skyline plots were inferred using the log-normal relaxed molecular clock model. Dotted vertical lines indicate one year and branches are scaled by time. The light grey line indicates the mean population diversity estimates through time, and the darker grey areas indicate the 95% HPD intervals of that estimate.
(PDF)

S7 Fig Demographic history of HIV-1 subjects. The maximum clade credibility tree and skyline plots are shown each subject group. Panels (a) to (i) represent subjects HIV1-9, respectively. See main text Fig 4 for more details. Trees and skyline plots were inferred using the log-normal relaxed molecular clock model. Dotted vertical lines indicate one year and branches are scaled by time. The light grey line indicates the mean population diversity estimates through time, and the darker grey areas indicate the 95% HPD intervals of that estimate.
(PDF)

S8 Fig The effect of undersampling on estimates of pairwise diversity and Tajima’s D from within host HCV sequence data. Three deep-sequenced datasets from Lu et al (2013), which represent three HCV subtype 1a infections (HCV isolates 1106, 1701, and 1706, respectively) were analysed to explore the potential effects of undersampling on estimating population genetic summary statistics. Specifically, we chose three genome regions of varying levels of diversity (the columns are ordered by increasing diversity, from left to right), where MPD indicates the mean pairwise diversity based on the full dataset. In each case, we generated 100 randomly subsampled datasets containing 5, 10, 12, 14, 16, 18, 20, 40, 60, 80, and 100 sequences. For each replicate, we estimated MPD and Tajima’s D in exactly the same way as for the real data. The red dashed lines correspond to the sample sizes used in the current study (n = 18 to n = 88). Panels A-C summarize the results for HCV isolates 1106, 1701, and 1709, respectively.
(PDF)

S9 Fig The effect of undersampling on estimates of pairwise diversity and Tajima’s D from within host HIV-1 sequence data. Appropriate HIV-1 datasets from Zanini et al (2016) and Dialdestoro et al (2016) were analysed to test the effects of sampling on estimating pairwise diversity and tajima’s D in HIV-1 within-host viral population. As in S8 Fig, three non-overlapping genomic regions were chosen, 350-400nt long. These genomic regions were selected to represent regions of low to high diversity, and each was required to have a minimum depth of 500 sequences. In each case, we generated 100 randomly subsampled datasets containing 5, 10, 12, 14, 16, 18, 20, 40, 60, 80, and 100 sequences. For each replicate, we estimated MPD and Tajima’s D in exactly the same way as for the real data. The red dashed lines correspond to the sample sizes used in the current study (n = 18 to n = 88). Panels A and B represent patients 1 and 3 (at timepoints 11 and 5, respectively), from Zanini et al (2016), while panel C represents patient 3 sampled at day 108 from Dialdestoro et al (2016).
(PDF)

S1 Table HCV Subjects. (DOCX)

S2 Table Primers for RT, PCR and sequencing. (DOCX)

S3 Table Primer combinations for amplification of HCV envelope. (DOCX)

S1 Text Sequencing information for treated HCV subjects. (DOCX)

S2 Text Example XML code to specify the skew-normal distributed molecular clock. (DOCX)
