# Supplementary figures and images for: Exceptional Heterogeneity in Viral Evolutionary Dynamics Characterises Chronic Hepatitis C Virus Infection
Source: PLoS Pathog. 2016 Sep 15;12(9):e1005894. doi: 10.1371/journal.ppat.1005894 (PMC5025083; doi:10.1371/journal.ppat.1005894)

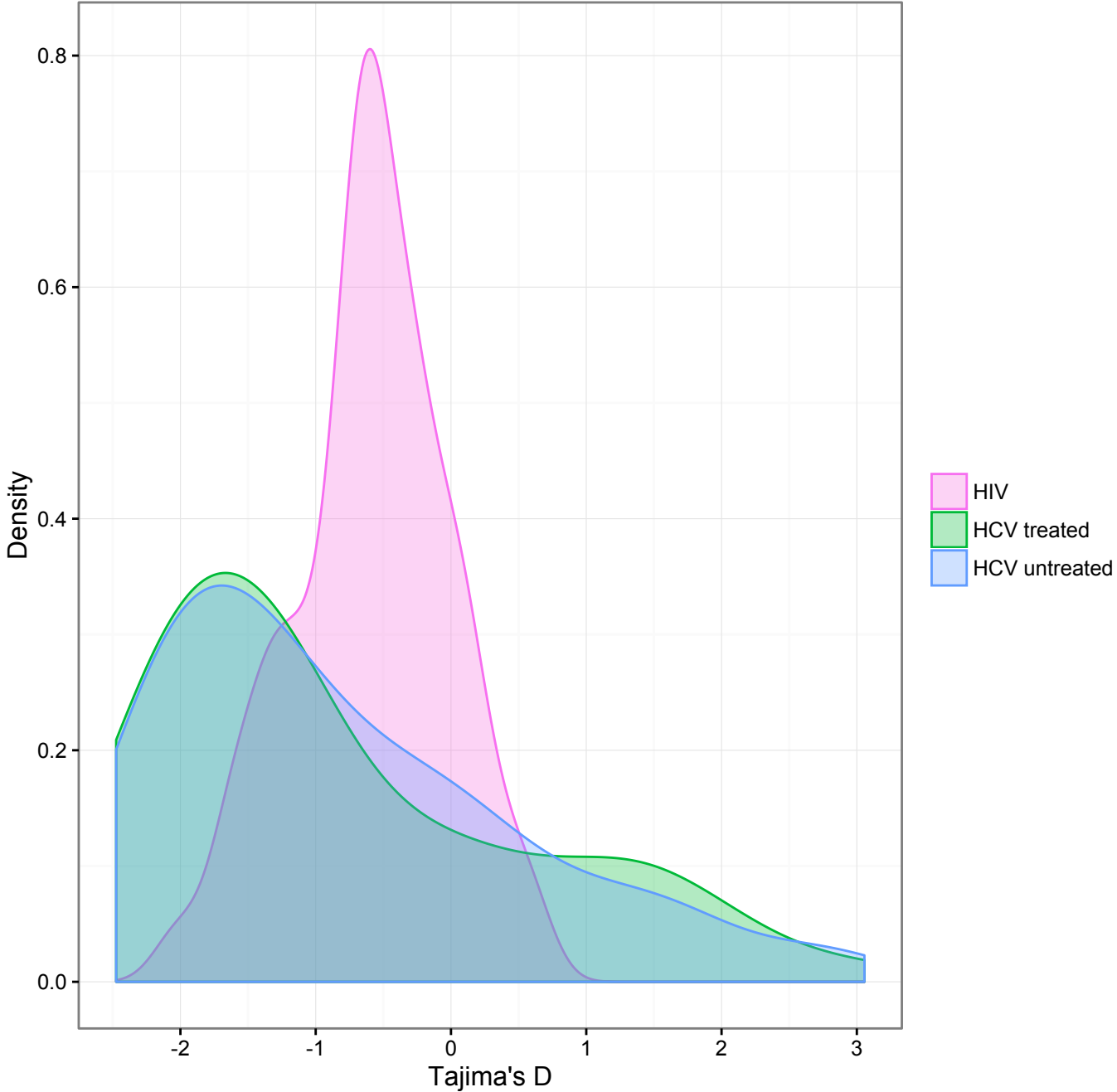

Supplement: S1 Fig — The distributions of Tajima’s D values are plotted for each cohort (HCV untreated, HCV treated, and HIV-1). The HCV untreated and treated groups have very similar distribution of Tajima’s D values, which show greater positive skew and variance compared to HIV-1 patients. (PDF) [file ppat.1005894.s001.pdf]

a) HCV untreated

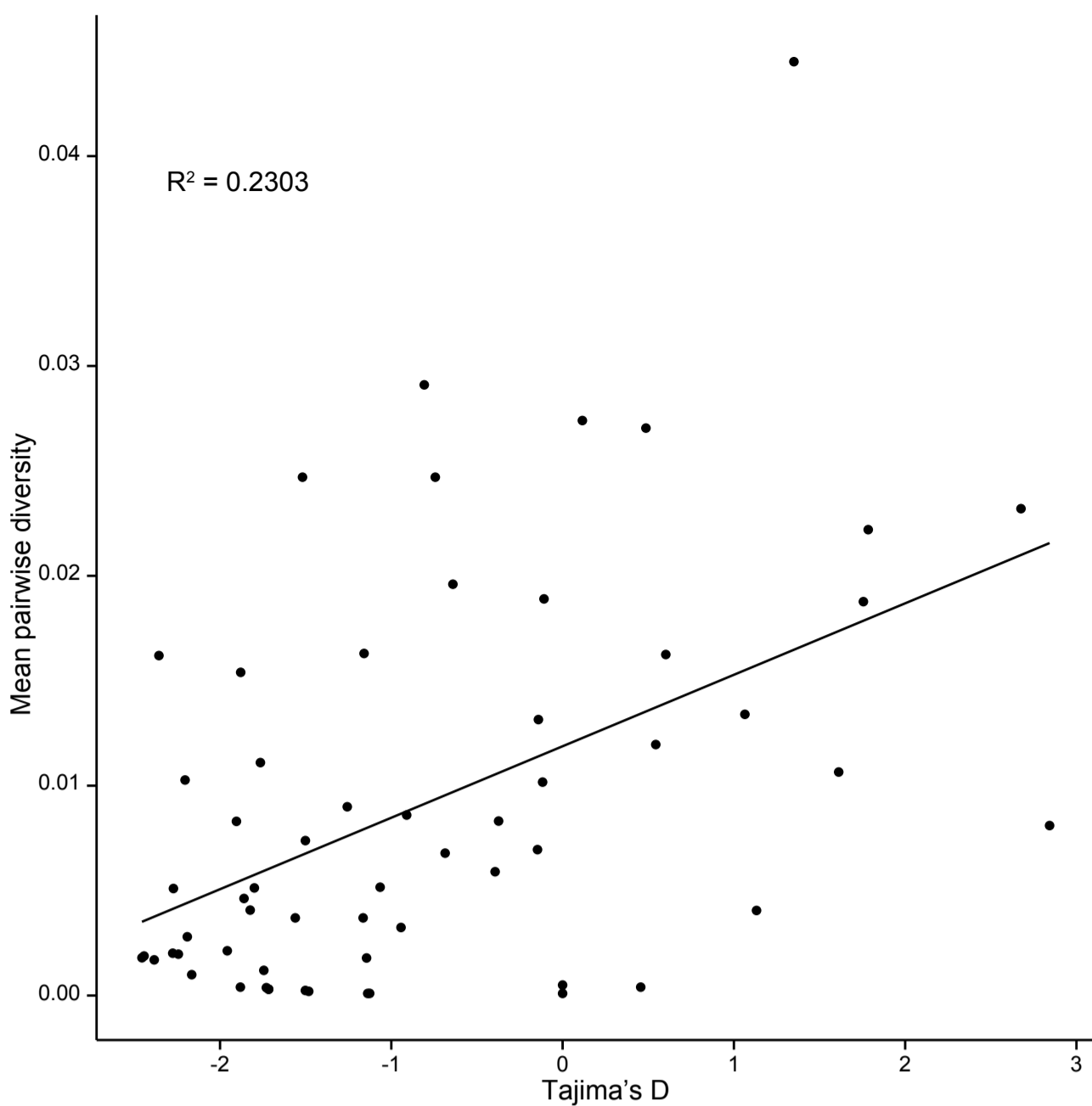

b) HCV treated

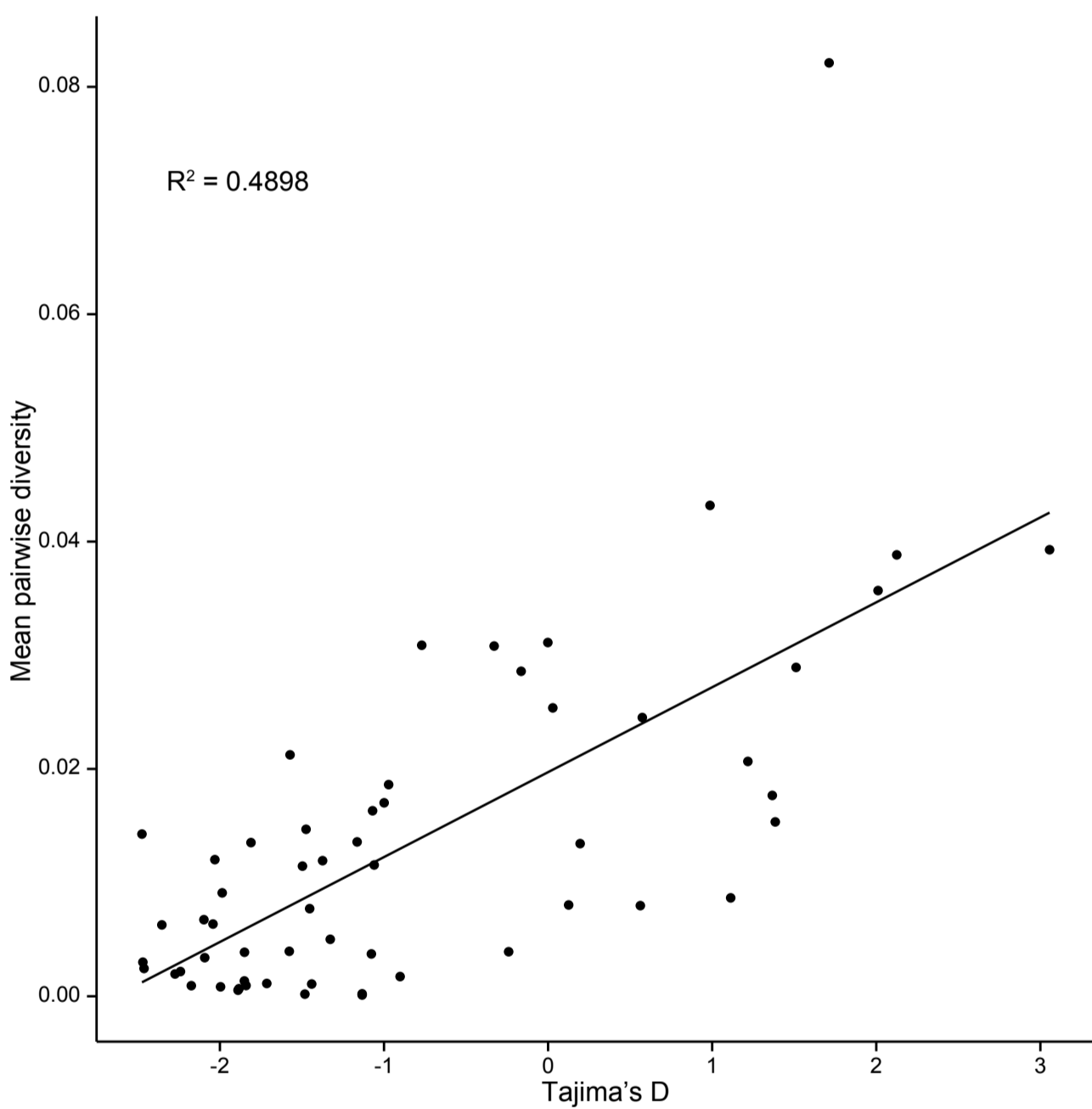

c) HIV

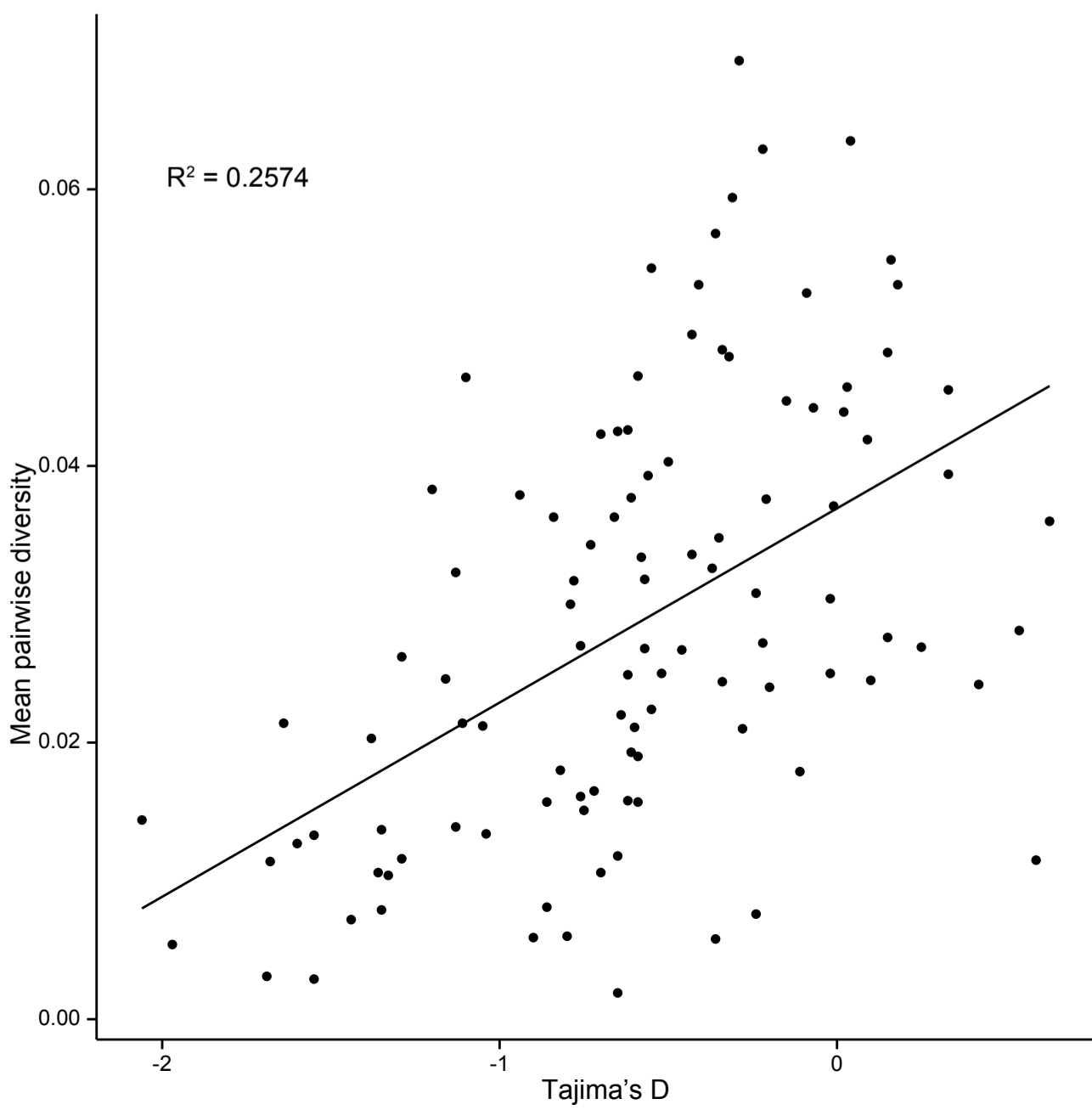

Supplement: S2 Fig — Tajima’s D (x-axis) is plotted against pair-wise nucleotide diversity (y-axis) for all time-points and subjects. (a) HCV untreated group; (b) HCV treated group; (c) HIV-1 group. A fitted linear regression model and associated R2 value are shown for each group. (PDF) [file ppat.1005894.s002.pdf]

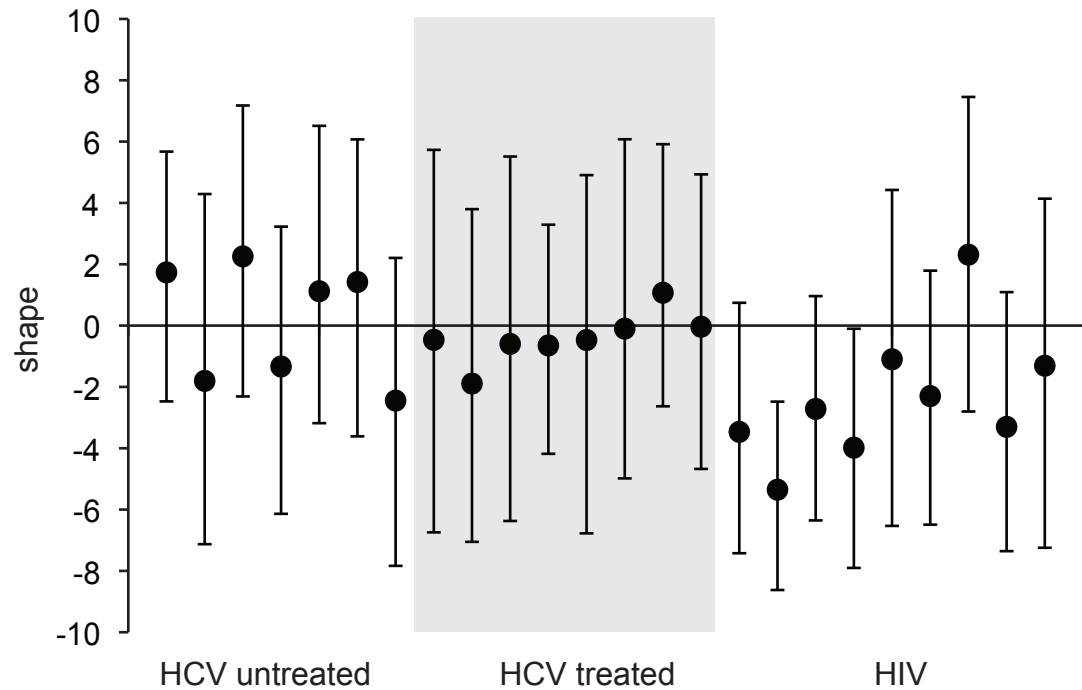

Supplement: S3 Fig — Mean estimate (filled circle) and 95% confidence intervals (vertical bars) of the shape parameter (y-axis) are shown for all subjects (x-axis). (PDF) [file ppat.1005894.s003.pdf]

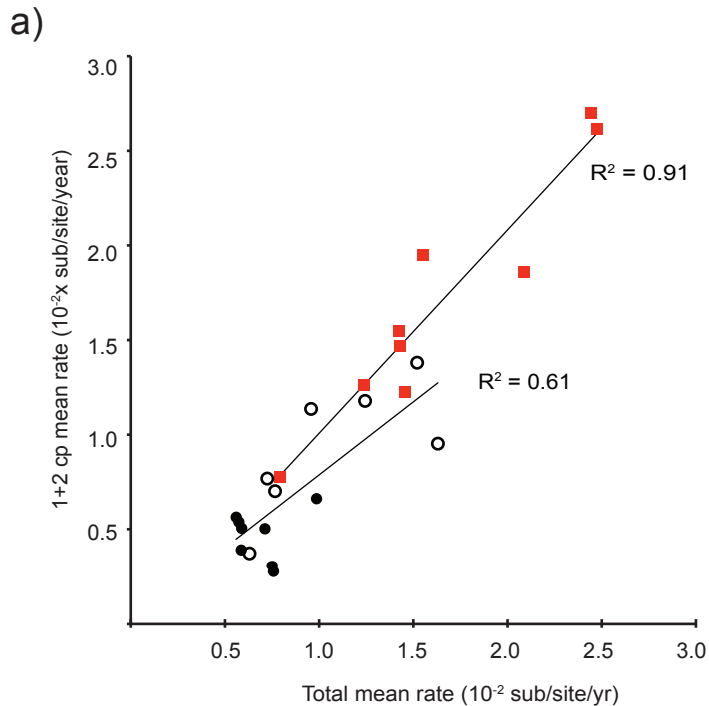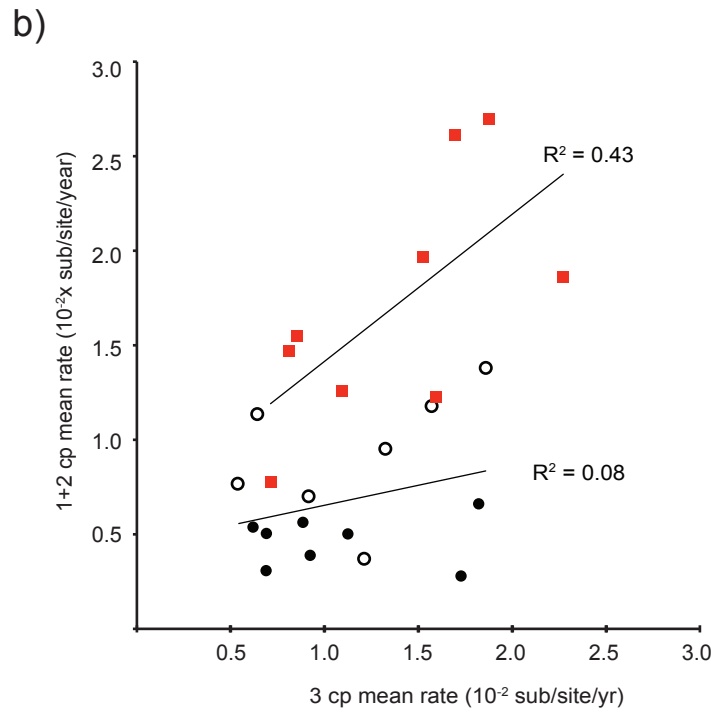

Supplement: S4 Fig — Mean estimate of the evolutionary rate for first and second codon positions (y-axis, both panels) is plotted against (a) the total mean evolutionary rate for all sites, and (b) the mean evolutionary rate for third codon positions. Each point represents a different subject. Subjects in the untreated HCV group are shown as open circles, those in the treated HCV group as filled circles, and those in the HIV-1 group as red squares. A fitted linear regression model and associated R2 value are shown for HCV and HIV-1 groups. (PDF) [file ppat.1005894.s004.pdf]

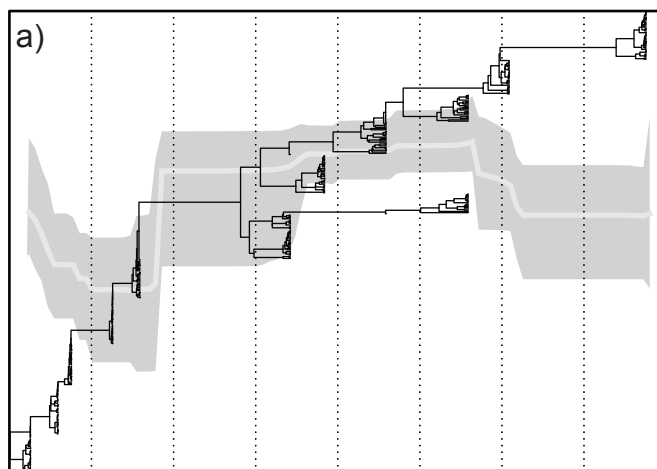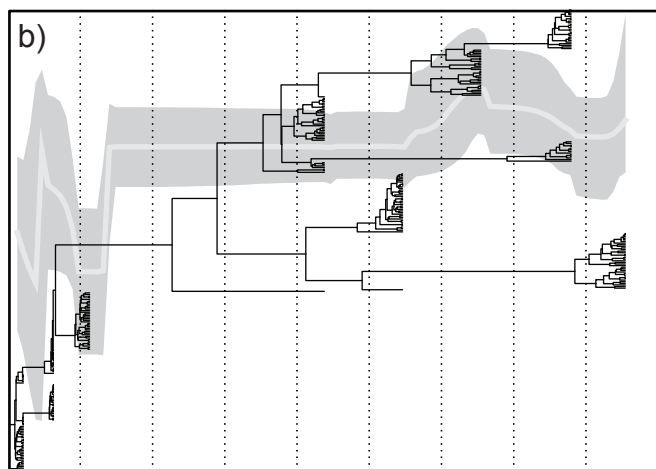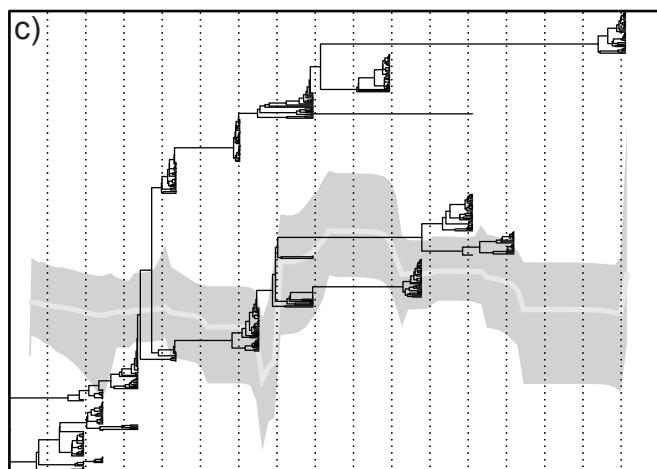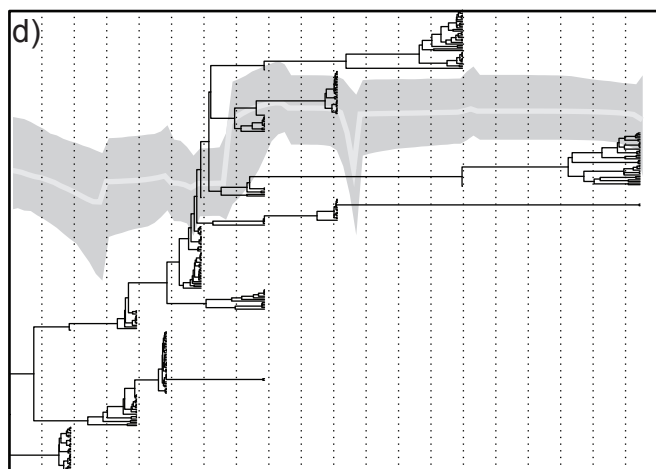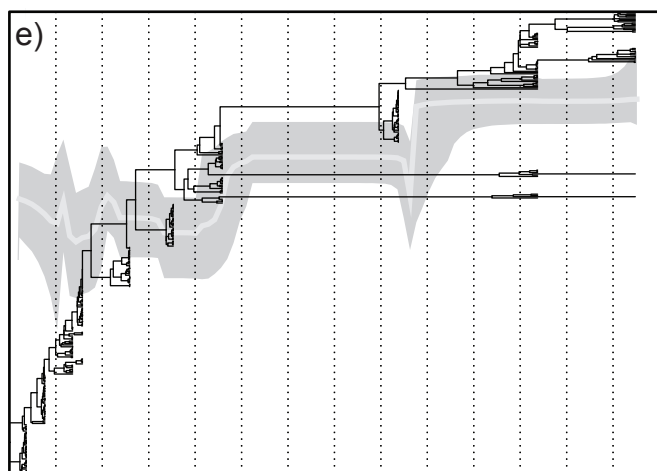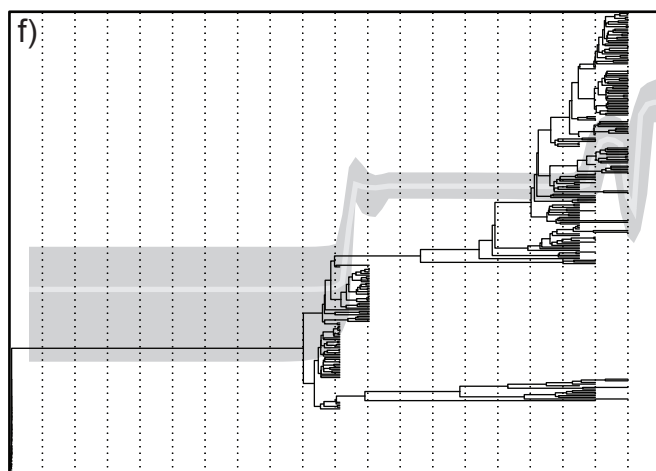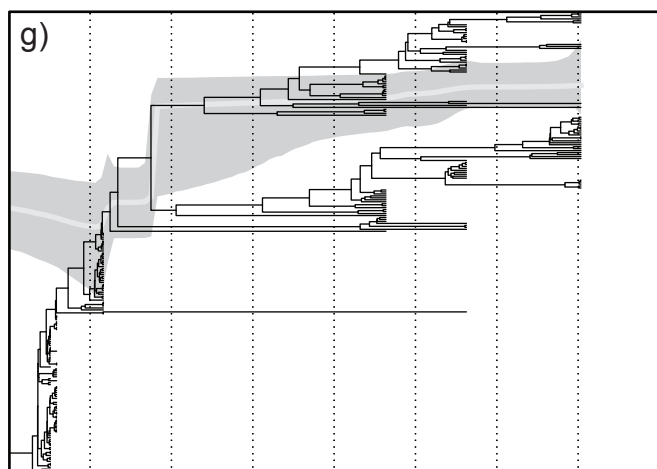

Supplement: S5 Fig — The maximum clade credibility tree and skyline plots are shown each subject group. Panels (a) to (g) represent subjects U1-7, respectively. See main text Fig 4 for more details. Trees and skyline plots were inferred using the log-normal relaxed molecular clock model. Dotted vertical lines indicate one year and branches are scaled by time. The light grey line indicates the mean population diversity estimates through time, and the darker grey areas indicate the 95% HPD intervals of that estimate. (PDF) [file ppat.1005894.s005.pdf]

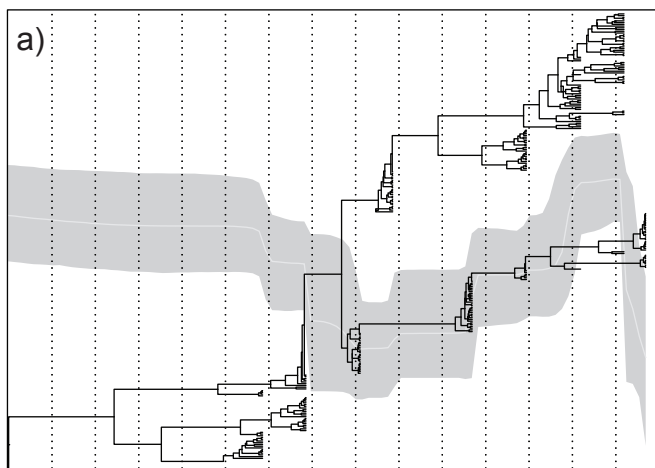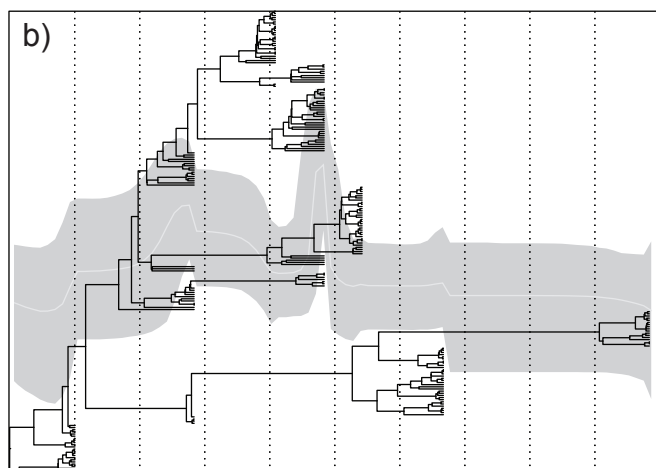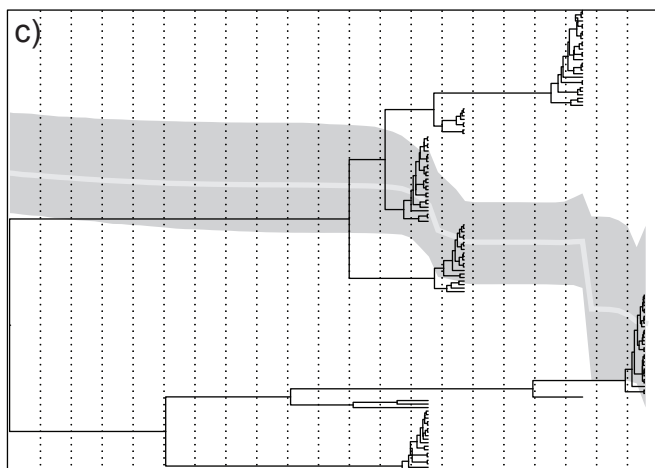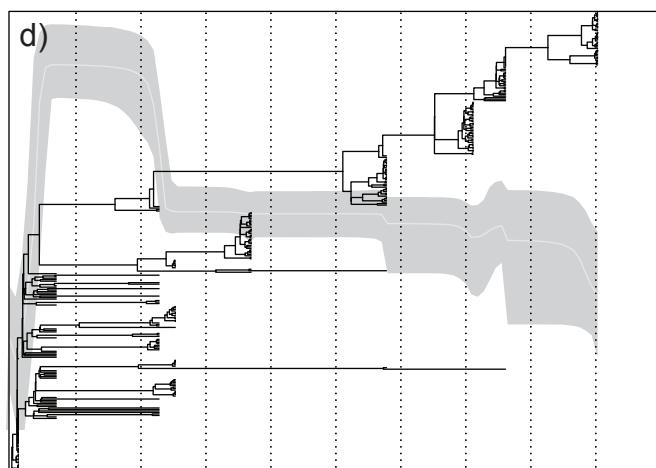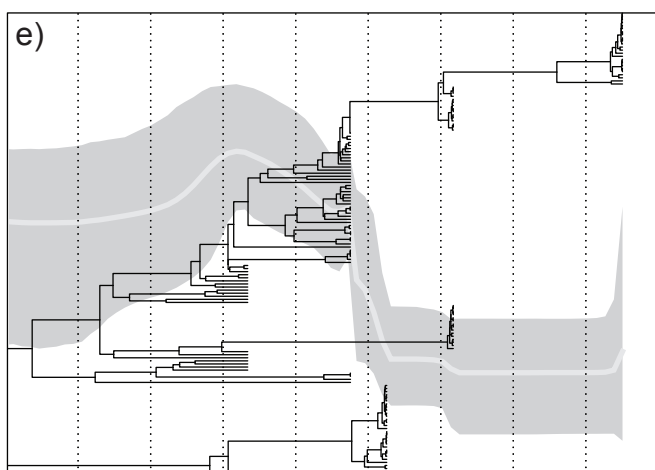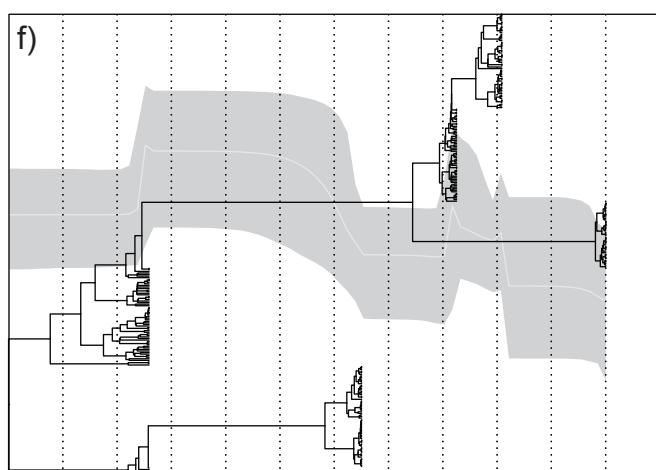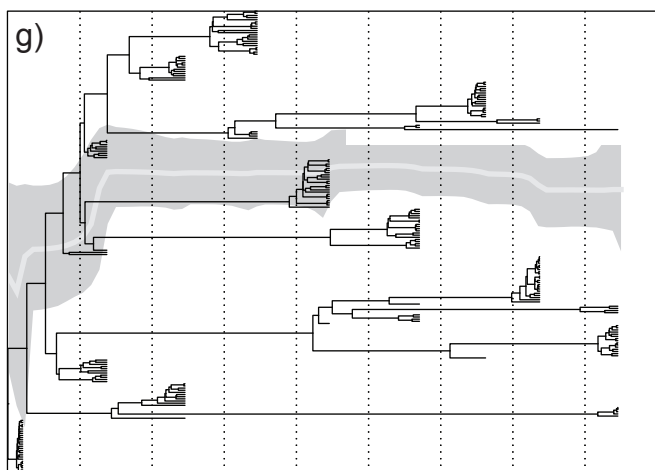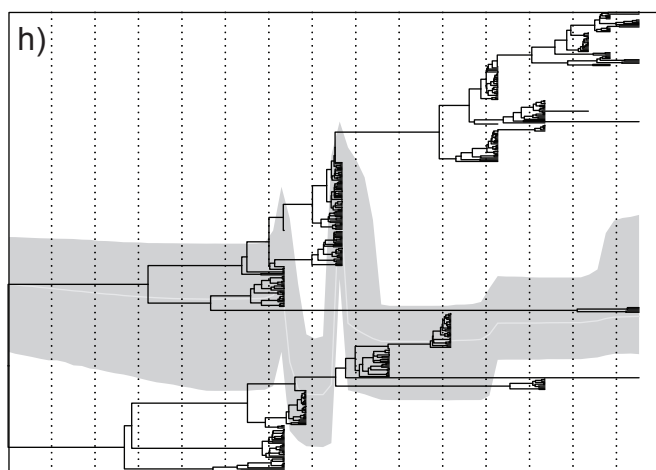

Supplement: S6 Fig — The maximum clade credibility tree and skyline plots are shown each subject group. Panels (a) to (h) represent subjects T1-8, respectively. See main text Fig 4 for more details. Trees and skyline plots were inferred using the log-normal relaxed molecular clock model. Dotted vertical lines indicate one year and branches are scaled by time. The light grey line indicates the mean population diversity estimates through time, and the darker grey areas indicate the 95% HPD intervals of that estimate. (PDF) [file ppat.1005894.s006.pdf]

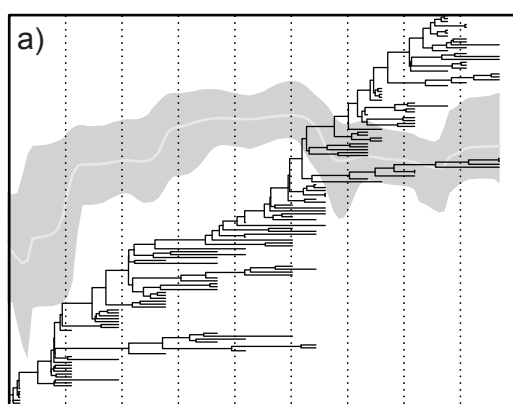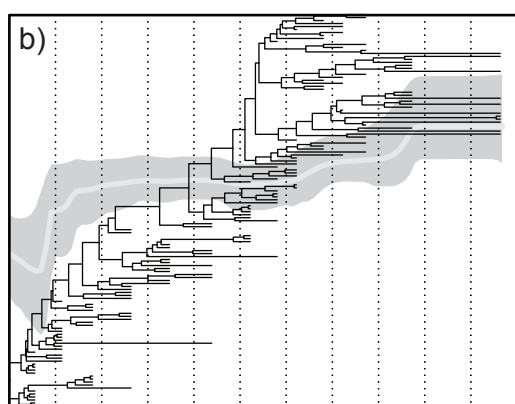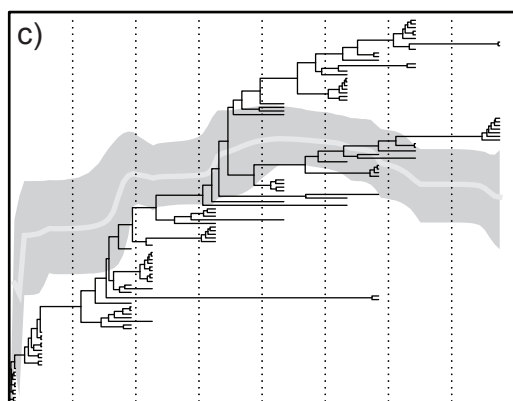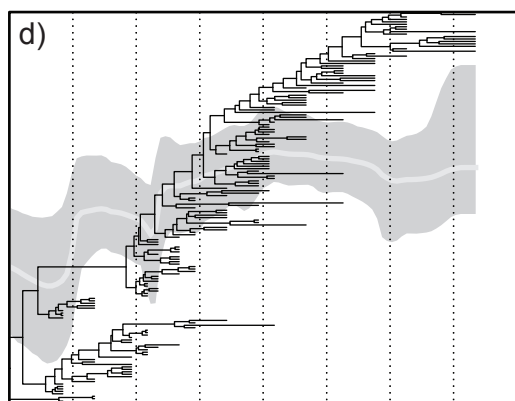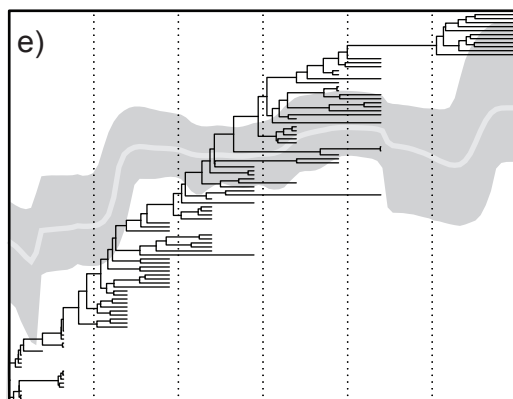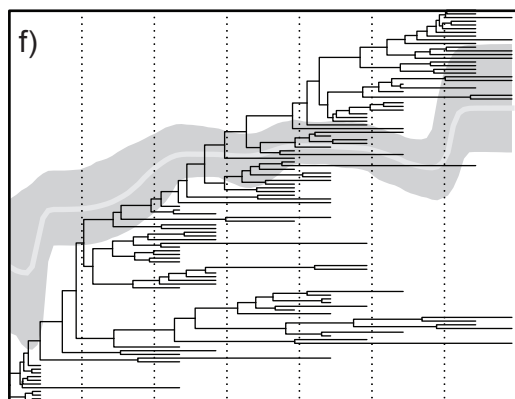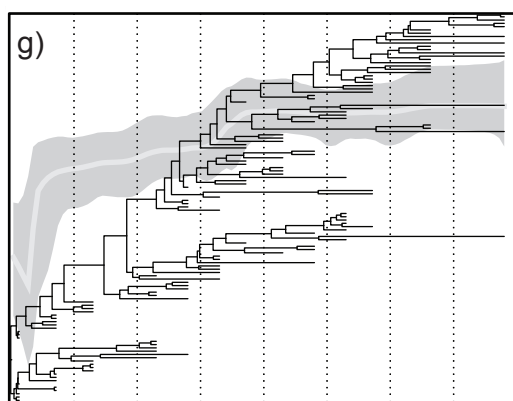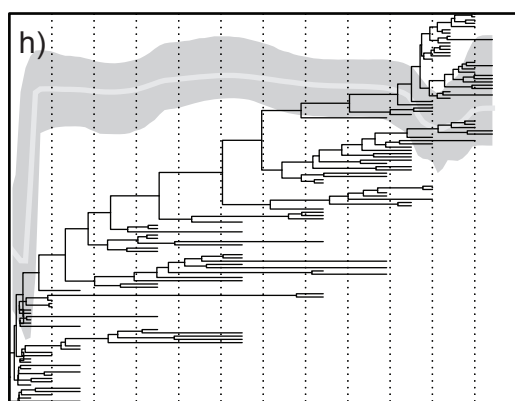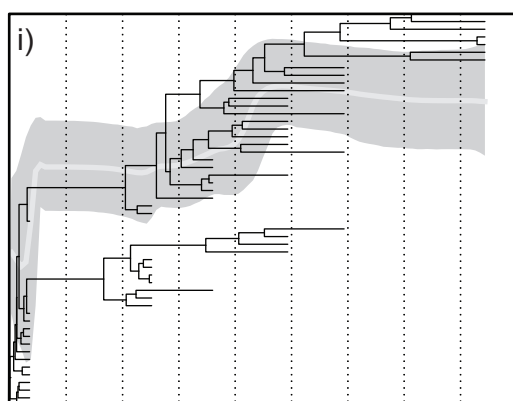

Supplement: S7 Fig — The maximum clade credibility tree and skyline plots are shown each subject group. Panels (a) to (i) represent subjects HIV1-9, respectively. See main text Fig 4 for more details. Trees and skyline plots were inferred using the log-normal relaxed molecular clock model. Dotted vertical lines indicate one year and branches are scaled by time. The light grey line indicates the mean population diversity estimates through time, and the darker grey areas indicate the 95% HPD intervals of that estimate. (PDF) [file ppat.1005894.s007.pdf]

# HIV

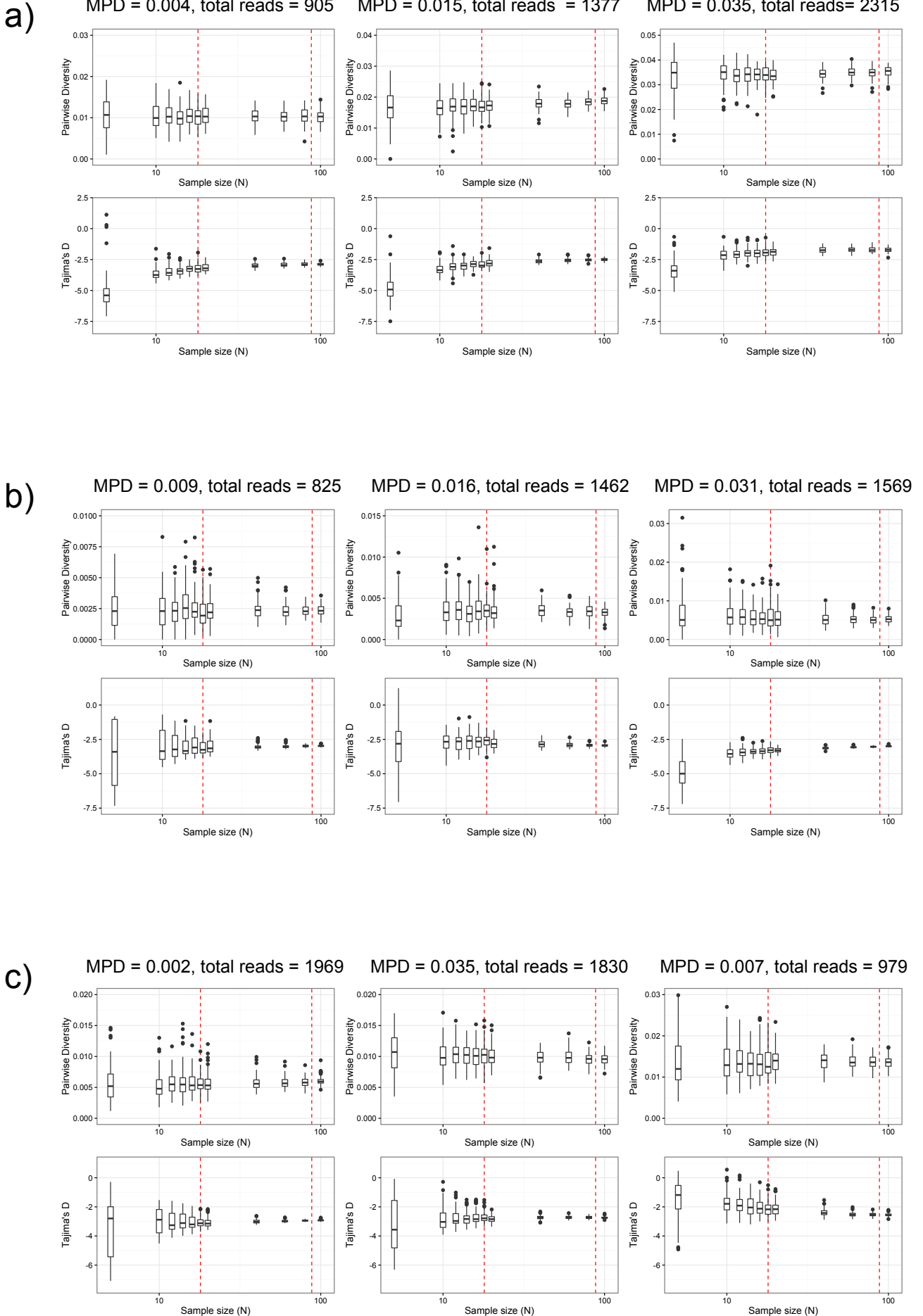

Supplement: S9 Fig — Appropriate HIV-1 datasets from Zanini et al (2016) and Dialdestoro et al (2016) were analysed to test the effects of sampling on estimating pairwise diversity and tajima’s D in HIV-1 within-host viral population. As in S8 Fig, three non-overlapping genomic regions were chosen, 350-400nt long. These genomic regions were selected to represent regions of low to high diversity, and each was required to have a minimum depth of 500 sequences. In each case, we generated 100 randomly subsampled datasets containing 5, 10, 12, 14, 16, 18, 20, 40, 60, 80, and 100 sequences. For each replicate, we estimated MPD and Tajima’s D in exactly the same way as for the real data. The red dashed lines correspond to the sample sizes used in the current study (n = 18 to n = 88). Panels A and B represent patients 1 and 3 (at timepoints 11 and 5, respectively), from Zanini et al (2016), while panel C represents patient 3 sampled at day 108 from Dialdestoro et al (2016). (PDF) [file ppat.1005894.s009.pdf]
