# Supplementary material for: Exceptional Heterogeneity in Viral Evolutionary Dynamics Characterises Chronic Hepatitis C Virus Infection
Source: PLoS Pathog. 2016 Sep 15;12(9):e1005894. doi: 10.1371/journal.ppat.1005894 (PMC5025083; doi:10.1371/journal.ppat.1005894)
Supplement: S8 Fig — Three deep-sequenced datasets from Lu et al (2013), which represent three HCV subtype 1a infections (HCV isolates 1106, 1701, and 1706, respectively) were analysed to explore the potential effects of undersampling on estimating population genetic summary statistics. Specifically, we chose three genome regions of varying levels of diversity (the columns are ordered by increasing diversity, from left to right), where MPD indicates the mean pairwise diversity based on the full dataset. In each case, we generated 100 randomly subsampled datasets containing 5, 10, 12, 14, 16, 18, 20, 40, 60, 80, and 100 sequences. For each replicate, we estimated MPD and Tajima’s D in exactly the same way as for the real data. The red dashed lines correspond to the sample sizes used in the current study (n = 18 to n = 88). Panels A-C summarize the results for HCV isolates 1106, 1701, and 1709, respectively. (PDF) [file ppat.1005894.s008.pdf]

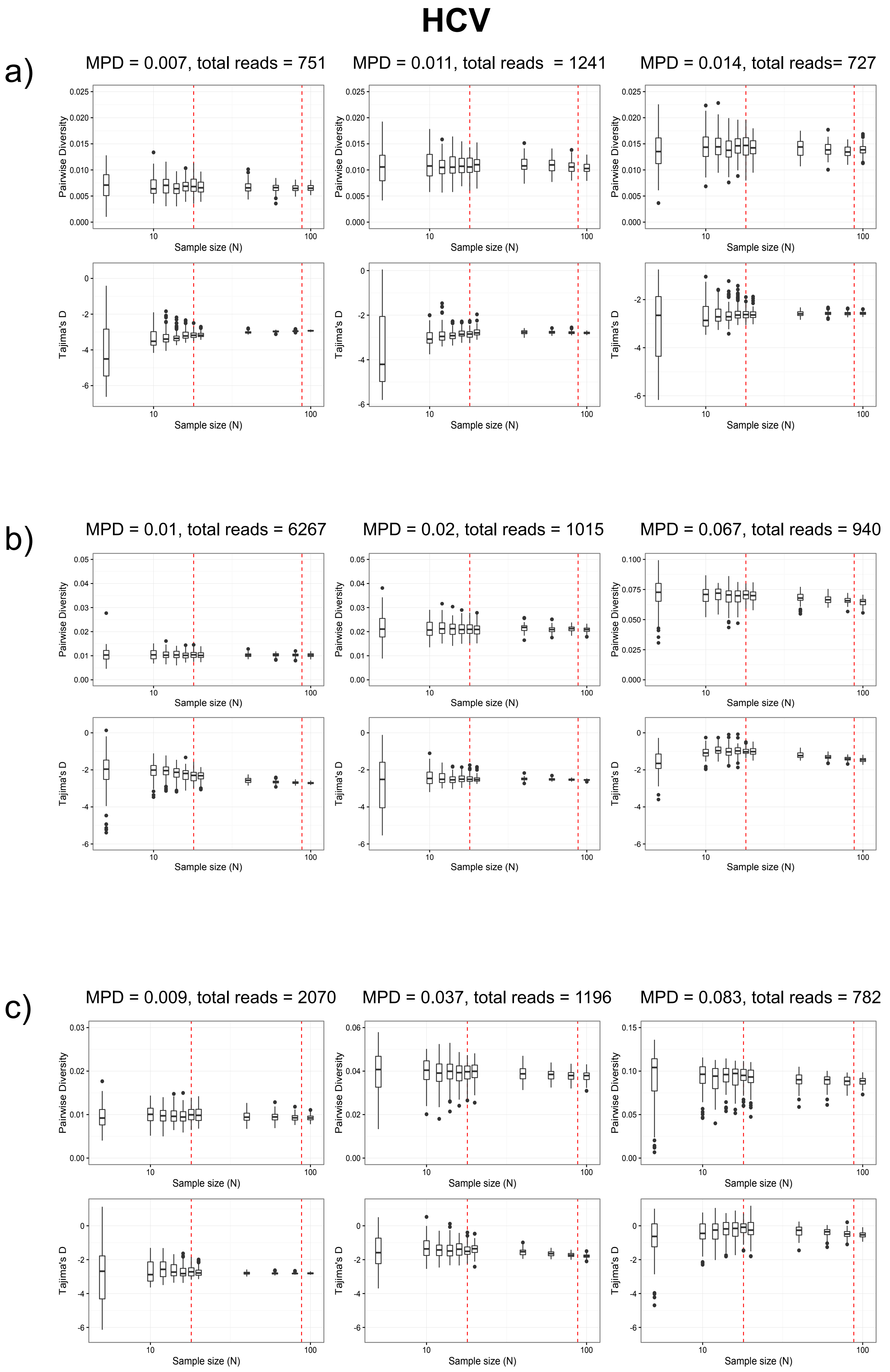
HCV
a)
MPD = 0.007, total reads = 751
MPD = 0.011, total reads = 1241
MPD = 0.014, total reads= 727
b)
MPD = 0.01, total reads = 6267
MPD = 0.02, total reads = 1015
MPD = 0.067, total reads = 940
c)
MPD = 0.009, total reads = 2070
MPD = 0.037, total reads = 1196
MPD = 0.083, total reads = 782
Pairwise Diversity
Tajima's D
Sample size (N)
10
100
