# Supplementary material for: Exceptional Heterogeneity in Viral Evolutionary Dynamics Characterises Chronic Hepatitis C Virus Infection
Source: PLoS Pathog. 2016 Sep 15;12(9):e1005894. doi: 10.1371/journal.ppat.1005894 (PMC5025083; doi:10.1371/journal.ppat.1005894)
Supplement: S1 Table — (DOCX) [file ppat.1005894.s010.docx]

**Table S1. HCV Subjects**

| Subject (this study) | Subject (previous study) | Number of sequences used in analysis | Length  (nt) | Number of sampling times | HCV subtype | Time span of samples (years) | Source |
| --- | --- | --- | --- | --- | --- | --- | --- |
| U1 | 1 | 374 | 528 | 11 | 4D | 7.75 | Farci *et al* (2006) |
| U2 | 4 | 288 | 528 | 8 | 1B | 8.67 | Farci *et al* (2006) |
| U3 | 11 | 418 | 528 | 12 | 1A | 15 | Farci *et al* (2006) |
| U4 | 1 | 287 | 528 | 8 | 1B | 18.92 | Farci *et al* (2012) |
| U5 | 2 | 366 | 528 | 10 | 1B | 14.17 | Farci *et al* (2012) |
| U6 | 3 | 278 | 528 | 6 | 1A | 23.25 | Farci *et al* (2012) |
| U7 | 5 | 235 | 528 | 6 | 1A | 7.42 | Farci *et al* (2012) |
| T1 | NA | 266 | 480 | 9 | 1B | 8.83 | This study |
| T2 | NA | 216 | 480 | 7 | 1B | 8.83 | This study |
| T3 | NA | 132 | 480 | 4 | 1B | 7 | This study |
| T4 | NA | 308 | 480 | 9 | 1B | 8.92 | This study |
| T5 | NA | 151 | 480 | 5 | 1B | 5.17 | This study |
| T6 | NA | 297 | 480 | 5 | 1B | 8.42 | This study |
| T7 | NA | 215 | 480 | 9 | 1B | 8.25 | This study |
| T8 | NA | 395 | 480 | 10 | 1B | 8.17 | This study |
