# Supplementary material for: Exceptional Heterogeneity in Viral Evolutionary Dynamics Characterises Chronic Hepatitis C Virus Infection
Source: PLoS Pathog. 2016 Sep 15;12(9):e1005894. doi: 10.1371/journal.ppat.1005894 (PMC5025083; doi:10.1371/journal.ppat.1005894)
Supplement: S2 Table — (DOCX) [file ppat.1005894.s011.docx]

**Table S2 Primers for RT, PCR and sequencing**

All listed 5’-3’; locations based on HCV reference sequence AF009606.

| Name | Sequence | Location |
| --- | --- | --- |
| **Forward:** |  |  |
| 5'UTR ExF 1 | CCCTGTGAGGAACTWCTGTCTTCACGC | 43-96 |
| 5'UTR InF 3 | TCTAGCCATGGCGTTAGTRYGAG | 78-100 |
| E1 ExF | TATGCAACAGGGAATTTGCCC | 831-851 |
| E1 InF | TTGACCATCCCAGCTTCCG | 894-912 |
| H4 ExF | CGCATGGCATGGGACATGAT | 1290-1309 |
| H4 InF | GGGACATGATGATCAACTGG | 1300-1310 |
| B F | CTTGGGATATGATGATGAACTGG | 1297-1319 |
| C-strain1-F | TCTCGTGAGCATCTTTTCCC | 1536-1555 |
| C-strain2-F | CACGTCCCTCTTCACATCT | 1539-1557 |
| E2 ExF | AACACCAATGGCAGTTGGCA | 1584-1603 |
| E2 InF | CACATCAACAGGACTGCC | 1602-1619 |
| C F | AACTCGTCCGGATGCCCAGAGCG | 1683-1705 |
| D F | ACGGACTGCTTTCGGAAGCACCC | 2086-2110 |
| E F | TCAAATGGGAGTATGTCGTGTTGC | 2482-2505 |
| M13 F | GTAAAACGACGGCCAG | vector |
|  |  |  |
| **Reverse:** |  |  |
| 5'UTR ExR 2 | GGTGCACGGTCTACGAGACCT | 321-341 |
| 5'UTR InR 4 | CACTCGCAAGCACCCTATCAGGCAGT | 288-313 |
| V R | ACCATGTCCACGACGGCTTGTG | 1365-1384 |
| C-strain1-R | GTCCGAGCTCCTAGACTC | 1768-1785 |
| C-strain2-R | GAGGGTCCGGCATTATAATC | 1768-1787 |
| W R | CGGTCGAGGCGCGTAGTGCC | 1801-1820 |
| H4 InR | GTGAAGCAATACACCGGACCACA | 1848-1870 |
| H4 ExR | GGAGTGAAGCAATACACCGG | 1854-1873 |
| H4 ExR2 | GGGGTGAAACAATACACAGG | 1854-1873 |
| X R2 | CARTTGCACGCGGCTTCGAGCC | 2257-2278 |
| X R | CCTCGAGTCCAATTGCAT | 2270-2287 |
| Y R | AAGCGGCCTCAGCCTGAGCTA | 2563-2583 |
| E2 InR2 | TTATAGTATGGTGACAAGGTC | 2828-2848 |
| E2 ExR2 | TATTGTAACCACCATATGAGC | 2864-2884 |
| RT1 | CCGGTTCATCCAYTGC | 6095-6080 |
| M13 R | CAGGAAACAGCTATGAC | vector |
