# Supplementary material for: Exceptional Heterogeneity in Viral Evolutionary Dynamics Characterises Chronic Hepatitis C Virus Infection
Source: PLoS Pathog. 2016 Sep 15;12(9):e1005894. doi: 10.1371/journal.ppat.1005894 (PMC5025083; doi:10.1371/journal.ppat.1005894)
Supplement: S3 Table — (DOCX) [file ppat.1005894.s012.docx]

**Table S3 Primer combinations for amplification of HCV envelope**

| **Primary** | **Product (bp)** | **Secondary** | **Product (bp)** |
| --- | --- | --- | --- |
| 5'UTR 1 + 5'UTR 3 | 31-329 | 5'UTR 4 78-313 | 78-313 |
|  |  |  |  |
| E1 ExF + E2 ExR2 | 831-2884 | E1 InF +E2 InR2 | 894-2848 |
|  |  | H4 InF + H4 InR | 1300-1870 |
|  |  | B F + W R | 1297-1816 |
|  |  |  |  |
| E2 ExF + E2 ExR2 | 1584-2884 | E2 InF + E2 InR2 | 1602-2848 |
|  |  |  |  |
| E1 ExF + H4 ExR2 | 831-1873 | E1 InF + H4 InR | 894-1870 |
|  |  | H4 InF + H4 InR | 1300-1870 |
|  |  | B F + W R | 1297-1816 |
|  |  |  |  |
| H4 ExF + H4 ExR | 1290-1873 | H4 InF + H4 InR | 1300-1870 |
|  |  |  |  |
| H4 ExF + H4 ExR2 | 1290-1873 | H4 InF + H4 InR | 1300-1870 |
|  |  | B F + W R | 1297-1816 |
