# Supplementary material for: Exceptional Heterogeneity in Viral Evolutionary Dynamics Characterises Chronic Hepatitis C Virus Infection
Source: PLoS Pathog. 2016 Sep 15;12(9):e1005894. doi: 10.1371/journal.ppat.1005894 (PMC5025083; doi:10.1371/journal.ppat.1005894)
Supplement: S1 Text — (DOCX) [file ppat.1005894.s013.docx]

**S1 Text: Sequencing information for treated HCV subjects**

Both acutely and persistently infected patient samples were collected over a period of 12 years from Bari, Italy. Viral RNA was extracted from 140 μl of infected plasma using the QIAamp Viral RNA kit (Qiagen) and re-suspended in 60 μl dH20. Viral RNA was reverse transcribed to produce a single stranded cDNA using either avian myeloblastosis virus (AMV) RT (Promega) or Superscript (SS) II (Invitrogen). Multiple different primer pairs that spanned the HVR1 region were used to reduce template bias. The full set of primers used are available in Tables (S1 and S2). The Roche Expand High Fidelity PCR system was used in 2 rounds of nested PCR to amplify the desired region whilst minimising error. This is an enzyme blend containing both Taq polymerase and a proofreading polymerase. The PCR mix was prepared on ice according to the protocol (35.25 μl of dH_2_0, 5μl of 10x buffer, 1.5μl each of 10μM forward and reverse primers, 1μl dNTPs, 0,75μl Expand enzyme). In the first round of amplification, the reaction mix was added to 5μl cDNA to make a final volume of 50μl; 5μl of the primary PCR was then used as a template for the second round. Cycling conditions are as follows; in bold are those that were changed according to the primers and template to be amplified:

1. 94°C /2 min (denaturation)

2. 94°C /30 sec (denaturation)

3. **50** °**C** /30 sec (annealing)

4. 72°C/**60 sec** (elongation)

5. Repeat steps 2-4 another 34 times

6. 72°C/7 min (final elongation)

Purified PCR products were ligated into a plasmid vector (TOPO-TA Cloning kit, Invitrogen), and then used to transform competent *Escherichia coli* cells. The bacterial culture was spread on ampicillin agar plates (ampicillin concentration 100μg/ml, Sigma, UK) and incubated overnight at 37° to allow growth of bacterial colonies. Incorporation of the insert into the plasmid disrupts transcription of the ampicillin resistance gene, enabling selection of clones containing the product. Individual bacterial colonies were cultured overnight in 1ml of LB medium containing ampicillin (concentration 100μg/ml) in a 96-well plate system (Montage Plasmid Miniprep96 Kit, Millipore, UK). The supplied protocol was modified to allow use of a centrifuge rather than a vacuum manifold. Plasmid DNA was resuspended in 50μl of dH20. Successful transformation was confirmed by digestion using the enzyme EcoR1 (New England Biolabs, US), which cuts the vector at two sites located on either side of the insert, generating 2 linear strands of DNA. Digested products were run on a 1% agarose gel stained with ethidium bromide and visualised under UV light.

Envelope products were sequenced using ABI BigDye Terminator v3.0 system (Applied Biosciences, UK). Cycling conditions were as follows: 30 sec at 96°C, followed by 30 sec at 96°C, 15 sec at 50°C, and 4 min at 60°C (steps 2-4 were repeated 20 times). For inserts of <600bp, the M13 forward and reverse primers only were used; these primers bind specifically to the vector, allowing the whole insert to be sequenced in both directions. For larger inserts, additional internal primers were required to ensure that the entire length could be read successfully; in these cases, PCR primers located inside the product were used. Sequencing products were purified to remove unincorporated dye terminators by precipitation using 100% ethanol/sodium acetate, then washed twice with 70% ethanol. Dry pellets were sequenced on the ABI 3700 sequencer. Forward and reverse trace files were checked for congruency using the Staden software package (<http://staden.sourceforge.net>). Sequence data was aligned manually using the program SE-AL (http://evolve.zoo.ox.ac.uk), with terminal primer binding sites and any stop codons removed before analysis. Sequences are available at GenBank accession numbers KX111382 - KX113360.
