# Supplementary material for: Exceptional Heterogeneity in Viral Evolutionary Dynamics Characterises Chronic Hepatitis C Virus Infection
Source: PLoS Pathog. 2016 Sep 15;12(9):e1005894. doi: 10.1371/journal.ppat.1005894 (PMC5025083; doi:10.1371/journal.ppat.1005894)
Supplement: S2 Text — (DOCX) [file ppat.1005894.s014.docx]

**S2 Text: Example XML code to specify the skew-normal distributed molecular clock**

Specifying the molecular clock:

<arbitraryBranchRates id="branchRates" exp="true" centerAtOne="false">

<treeModel idref="treeModel"/>

<rates>

<parameter id="branchRates.rates" dimension="245" value="-9"/>

</rates>

</arbitraryBranchRates>

<distributionLikelihood id="branchRates.prior">

<distribution>

<skewNormalDistributionModel>

<location>

<parameter id="rate.prior.location" value="0" lower="-Infinity"/>

</location>

<scale>

<parameter id="rate.prior.scale" value="1" lower="0.0"/>

</scale>

<shape>

<parameter id="rate.prior.shape" value="0"/>

</shape>

</skewNormalDistributionModel>

</distribution>

<data>

<parameter idref="branchRates.rates"/>

</data>

</distributionLikelihood>

Operators:

<scaleOperator scaleFactor="0.75" weight="3">

<parameter idref="rate.prior.scale"/>

</scaleOperator>

<randomWalkOperator windowSize="0.1" weight="3">

<parameter idref="rate.prior.location"/>

</randomWalkOperator>

<randomWalkOperator windowSize="0.1" weight="3">

<parameter idref="rate.prior.shape"/>

</randomWalkOperator>

Priors:

<exponentialPrior mean="1">

<parameter idref="rate.prior.scale"/>

</exponentialPrior>

<normalPrior mean="0.0" stdev="100">

<parameter idref="rate.prior.location"/>

</normalPrior>

<normalPrior mean="0.0" stdev="3">

<parameter idref="rate.prior.shape"/>

</normalPrior>
